# Multiobjective adaptive predictive virtual synchronous generator control strategy for grid stability and renewable integration

**DOI:** 10.1038/s41598-025-93721-y

**Published:** 2025-03-18

**Authors:** Mrinal Kanti Rajak, Rajen Pudur

**Affiliations:** Department of Electrical Engineering, National Institute of Technology Arunachal Pradesh, Yupia, Jote, 791113 Arunachal Pradesh India

**Keywords:** Adaptive control, Multi-objective optimization, Predictive control, Renewable energy integration, Virtual synchronous generator, Smart grid technology, Engineering, Electrical and electronic engineering

## Abstract

A novel Adaptive Predictive Virtual Synchronous Generator (AP-VSG) control strategy is proposed for enhanced grid stability and seamless renewable energy integration. The method introduces adaptive inertia and damping mechanisms combined with multi-objective predictive optimization, specifically designed for parallel-connected Self-Excited Induction Generators (SEIGs). Unlike conventional approaches requiring multiple DC conversion stages, the proposed system implements parallel operation directly in the AC domain, reducing system complexity and conversion losses. The AP-VSG control incorporates a real-time adaptation of virtual inertia (H) ranging from 1 to 4 s and damping coefficient (D) from 20 to 65 pu, responding to grid frequency deviations and Rate of Change of Frequency (RoCoF). Experimental validation with parallel-connected 2.2 kW and 5.5 kW SEIGs demonstrates a 56% reduction in maximum RoCoF (from ± 0.48 Hz/s to ± 0.21 Hz/s), 33% improvement in frequency nadir (50.85–50.87 Hz), and 41% enhancement in damping ratio. Under fault conditions, the system maintains a current limit of 1.5 pu while providing reactive support up to 0.8 pu. The multi-objective optimization framework achieves 36.7% reduction in control effort while maintaining stability margins (PM$>45^{\circ }$, GM>6 dB). Statistical analysis confirms 95th percentile frequency regulation enhancement of 43.5% compared to conventional VSG control. The fault ride-through capability demonstrates voltage recovery within 100 ms with THD maintained below 3%. Experimental results verify robust performance under various grid disturbances, including voltage sags down to 0.2 pu and complete grid disconnection scenarios.

## Introduction

The global energy landscape is transforming significantly, driven by the urgent need for clean and sustainable energy sources^1,2^. As nations strive to meet ambitious renewable energy targets, integrating wind, solar, and other renewable energy sources into power grids has become essential for achieving long-term energy security and reducing greenhouse gas emissions. However, this shift towards a greener future presents critical challenges in maintaining grid stability and reliability. Traditional power grids, historically reliant on large synchronous generators, inherently benefit from the physical inertia. However, these generators provide to stabilize grid frequency. In contrast, renewable energy sources, particularly those interfaced via power electronic converters, lack this stabilizing inertia, making the grid more vulnerable to fluctuations and disturbances^3^.

Virtual Synchronous Generators (VSGs) have emerged as a promising solution to this challenge by mimicking the inertia and damping characteristics of synchronous machines^4–6^. Integrated with power electronic converters, VSGs provide synthetic inertia, enabling better frequency regulation in renewable-rich grids. While VSGs have shown potential in enhancing the integration of renewable energy sources, their performance can be further improved, especially in the face of increasing grid complexity and variability^7^.

Several studies have explored various aspects of VSG technology, including its concepts, control strategies, and applications. However, many research gaps and challenges remain unaddressed. D’Arco et al.^8^ offered a classification of VSG implementations, comparing them to microgrid droop controllers. However, they did not provide a thorough performance comparison of different VSG approaches, revealing a need for a standardized framework to evaluate VSGs across various systems. Similarly, Zhong et al.^9^ introduced synchronverters, designed to mimic synchronous generators, but focused more on theory than practical implementation. Bridging the gap between theoretical models and real-world applications remains a challenge. Bevrani et al.^10^ provided an overview of VSG concepts and control strategies but emphasized more research on VSG stability analysis and optimization. Developing advanced tools for analyzing the stability of systems with multiple VSGs is crucial, especially in complex grids with high renewable energy penetration. Chen et al.^11^ explored how VSGs can improve power quality, though their study focused on specific issues. A broader challenge lies in developing control strategies that address multiple power quality problems simultaneously, which would offer more comprehensive solutions for grid stability. Alipoor et al.^12^ introduced a novel VSG design with an alternating moment of inertia to enhance system stabilization, but the study lacked comparative analysis with other topologies. Optimizing this concept for different scenarios remains a key challenge. Liu et al.^13^ compared the dynamic characteristics of VSGs and droop-controlled inverters in distributed generation systems, but their study was limited to specific conditions. Developing adaptive control strategies that can optimize VSG performance across a wider range of disturbances is necessary to improve system resilience. Tamrakar et al.^14^ discussed the concept of virtual inertia and its role in VSG technology, but identified a gap in coordinating multiple virtual inertia-providing devices. Efficient coordination strategies for large-scale systems with multiple VSGs remain a significant challenge. Chen et al.^15^ proposed integrating DC microgrids as VSGs into AC grids, but their focus on a specific approach leaves room for exploring alternative methods. Developing standardized interfaces for seamlessly integrating DC microgrids into AC grids is vital for improving system interoperability. Mo et al.^16^ evaluated different VSG models but did not cover the full range of available implementations, highlighting the need for a comprehensive evaluation framework to assess VSG models across various performance metrics. Shi et al.^17^ presented a control strategy for microgrids using VSGs but did not address scalability, which is crucial for adapting control strategies to different microgrid configurations. Peng et al.^18^ explored stability challenges in systems dominated by power electronics and proposed solutions using VSGs, though more research is needed on the interaction between multiple VSGs and their collective impact on stability. Fang et al.^19^ proposed a distributed virtual inertia implementation but did not explore centralized or hierarchical approaches, revealing a need for robust communication protocols to coordinate distributed VSGs in large power systems. Li et al.^20^ introduced a self-adaptive control strategy for VSGs to improve frequency stability, but their study focused only on frequency, leaving other aspects of power system stability unaddressed. Developing multi-objective control strategies to handle various stability concerns is essential. Wang et al.^21^ investigated the inertial dynamics of VSG-controlled wind turbines, though the study was limited to doubly-fed induction generator (DFIG) turbines. Expanding VSG control strategies to cover a broader range of renewable energy sources and storage systems is a key challenge. Chen et al.^22^ examined VSG dynamic properties but did not offer a comprehensive analysis across different topologies, indicating the need for standardized methods to compare dynamic performance. Finally, D’Arco et al.^23^ presented a VSG implementation for smart grids but did not explore the potential of machine learning and artificial intelligence in VSG control. Incorporating these technologies could enhance VSG adaptability and performance in complex grid environments.

Recent advancements in Virtual Synchronous Generator (VSG) technology aim to address key challenges related to grid stability and frequency support, particularly in the context of renewable energy integration and microgrids. Several studies have contributed significant insights into optimizing VSG control strategies and improving their performance under various conditions.

Jia Liu et al.^24^ proposed a cost-efficient optimization procedure for VSG parameters, designed to minimize total energy dissipation (TED) while ensuring adequate grid frequency support. The authors introduced a systematic modelling approach and calculation algorithm for TED and Maximum Grid Frequency Deviation (MGFD) during frequency regulation transients. By considering MGFD as a constraint and TED as the optimization objective, the study developed a method for identifying optimal VSG parameters. Their findings also explored the influence of key VSG parameters on MGFD and TED, as well as the effects of VSG penetration on optimization results. X. Ding et al.^25^ presented a comprehensive review combining VSG control with advanced Deep Learning (DL) and Reinforcement Learning (RL) algorithms in the context of the energy internet (EI). They introduced the basic principles and classification of VSGs and then reviewed the development of DL and RL algorithms. The paper summarized recent research on VSG control incorporating these algorithms and identified major challenges and research trends. The review highlights the potential for machine learning techniques to improve VSG control, especially in complex, distributed energy systems like the EI. Bo Long et al.^26^ addressed the challenge of low-voltage fault ride-through in VSGs by introducing a virtual power compensation (VPC) strategy combined with an enhanced current-limiting method. The study analyzed the transient stability of VSGs under current-limiting conditions and revealed the mechanisms behind their instability. By examining phase portraits, the authors demonstrated how the proposed VPC strategy enhances transient stability. Additionally, a detailed quantitative analysis of control parameters and hardware-in-the-loop experiments validated the effectiveness of the proposed scheme in improving fault ride-through capability. Yulin Kuang et al.^27^ focused on improving pre-synchronization control strategies for grid-connected VSGs. Conventional methods often rely on phase-locked loops and nonlinear computations, which can lead to high hardware resource consumption and potential instability. To address this, the authors proposed a pre-synchronization control strategy based on simple linear calculations of voltage and angle differences between the VSG and the grid. Using PI controllers to process voltage and frequency differences, the proposed strategy achieved smooth grid connections. Experimental results confirmed its effectiveness in enhancing grid stability during synchronization. Jianwei Zhang et al.^28^ explored matrix converters (MC) as an interface for renewable power generation in microgrids. The MC-interfaced systems, like other power electronic converter-interfaced systems, lack the natural inertia and damping necessary to support grid frequency and voltage. To overcome this, the authors proposed a VSG control strategy tailored for MCs, incorporating secondary frequency control to restore nominal system frequency after load fluctuations. A synchronization control strategy based on virtual power was also introduced, and experimental validation demonstrated the correctness of the proposed methods. Alisher Askarov et al.^29^ investigated an alternative current-controlled VSG (CC-VSG) structure, which poses challenges due to its third-order complexity and lack of damping torque during grid strength variations. The authors proposed a feedforward controller to enhance the damping properties of the CC-VSG. By reducing the model order to second order through a closed-loop transfer function analysis, the study demonstrated that the enhanced CC-VSG structure offers improved stability and flexibility for achieving desired dynamic responses across different grid strengths and load conditions. Rui Liu et al.^30^ examined the behaviour of VSGs in low-voltage islanded multi-bus microgrids (LVIMB-MG), where interactions between VSGs can lead to low-frequency oscillations (LFOs). Previous analyses had neglected important factors such as the microgrid’s required rate of change of frequency (RoCoF) and steady-state active power-frequency (P-f) characteristics. The authors proposed a distributed low-frequency oscillation damping (DLFOD) control strategy to mitigate these oscillations by adjusting the VSG’s phase angle and active power setpoint. The approach was validated through theoretical analysis, real-time simulations, and experiments.

Finally, Waqar Tahir et al.^31^ focused on seamless switching between islanding and grid-connected modes for VSG inverters. Their modified control strategy demonstrated improved frequency transient response and reduced inrush current during synchronization from 120A to nominal levels. The proposed pre-synchronization technique outperformed traditional methods in terms of active power, reactive power, and frequency stability, ensuring smooth transitions from off-grid to on-grid operation.

Wang et al.^32^ introduced an adaptive control strategy for virtual synchronous generators (VSGs) to optimize the damping ratio, effectively mitigating power oscillations and enhancing dynamic response. Expanding upon this, Li et al.^33^ proposed a dual-adaptivity inertia control strategy that balances power and frequency regulation under varying operational conditions. Data-driven methodologies, such as reinforcement learning-based control by Yushuai Li et al.^34^, advanced VSG adaptability by dynamically adjusting parameters without relying on predefined system models. Similarly, Ding et al.^35^ employed deep reinforcement learning (DRL) to improve frequency stability and reduce response times in rapidly evolving grid scenarios. Lao et al.^36^ introduced a reliability-based adaptive inertia control strategy incorporating predictive mechanisms and multilevel consensus algorithms to enhance fault tolerance. Recent advancements summarized by Sadeque et al.^37^ provided a comprehensive overview of control innovations for grid-forming inverters and their integration into modern power systems. Additionally, Shadoul et al.^38^ reviewed VSG topologies and techniques, addressing the challenges of low-inertia systems and energy storage integration. Rehman et al.^39^ proposed a genetic algorithm-optimized VSG design, significantly improving photovoltaic system stability. Collectively, these studies illustrate the evolution from model-based to adaptive, data-driven control approaches, addressing scalability, computational efficiency, and fault tolerance, thereby paving the way for advanced grid-support functionalities in modern power systems.

Control theory, particularly Model Predictive Control (MPC), presents new opportunities to enhance Virtual Synchronous Generator (VSG) performance. MPC’s ability to manage multi-variable systems, respect operational constraints, and optimize performance over a prediction horizon makes it a promising approach for power system control. However, applying MPC to VSGs in the context of renewable energy integration and grid stability optimization is still in its early stages, and several open research questions remain.

**Figure 1:**
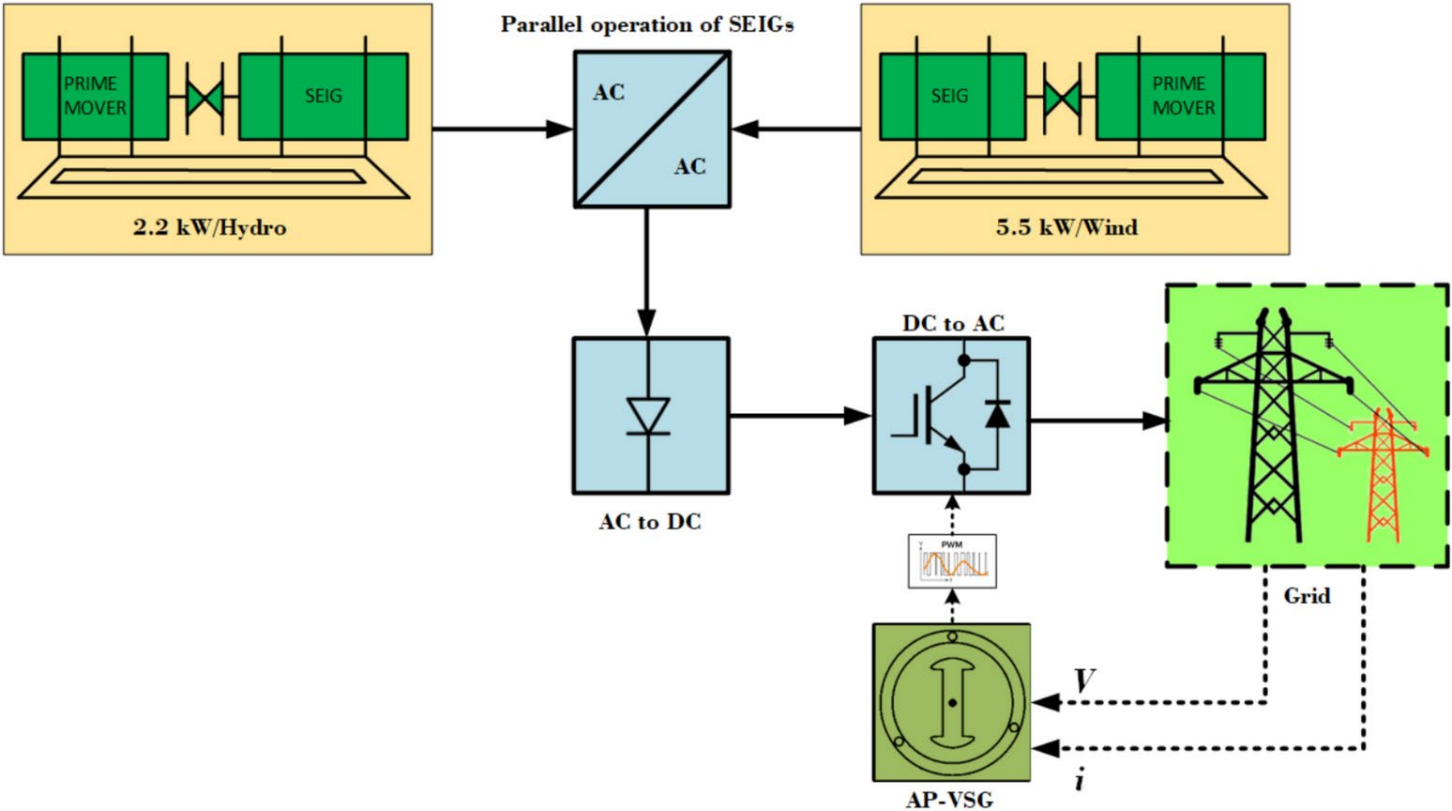
Block diagram of an AP-VSG with grid.

This paper introduces a novel control strategy: Adaptive Predictive Virtual Synchronous Generator (AP-VSG) Control, specifically tailored for parallel-connected Self-Excited Induction Generators (SEIGs), as shown in Fig. 1. The approach addresses the limitations of conventional Virtual Synchronous Generator (VSG) implementations by incorporating adaptive mechanisms and predictive control techniques, particularly in the management of multiple SEIGs operating in parallel. Unlike traditional methods, where generators are connected using multiple converters—each generator’s output is first converted to DC, paralleled, and then converted back to AC for grid connection—the proposed method enables parallel operation directly within the AC domain. This innovation reduces the number of required converters, thereby minimizing system complexity and lowering costs.

The AP-VSG control strategy is designed to optimize grid stability while facilitating seamless renewable energy integration. It utilizes a multi-objective framework to balance various system requirements, such as frequency regulation, voltage stability, and efficient power sharing. By directly managing the interactions and dynamics of parallel SEIGs within the AC domain, this control method enhances coordination and overall system performance, particularly in wind farms or other applications involving multiple induction generators. The proposed control not only improves individual SEIG performance but also ensures their collective operation contributes effectively to grid stability and power quality. Additionally, by reducing the number of power electronic stages, the approach enhances system efficiency and cost-effectiveness, providing a promising solution for large-scale renewable energy integration.

The significance of this research lies in its potential to enhance power system resilience and reliability amidst increasing renewable energy penetration. By improving the performance of VSGs, the AP-VSG control strategy can help overcome barriers to high renewable energy integration, supporting global efforts to transition to sustainable energy systems. Moreover, the multi-objective nature of the proposed control strategy allows for a holistic approach to grid management. This approach considers not only frequency and voltage stability but also power quality, energy efficiency, and equitable load sharing among distributed generators.

The originality of this work is multi-faceted. First, while adaptive control and MPC have both been applied separately to power systems, their synergistic integration in the context of VSGs represents a novel approach. The adaptive mechanisms allow the controller to adjust its behaviour in real-time based on changing grid conditions, while the predictive aspect enables proactive control actions that anticipate and mitigate potential instabilities.

Second, the multi-objective optimization framework developed in this research goes beyond traditional VSG control objectives. It incorporates a comprehensive set of performance metrics, including frequency stability, voltage regulation, power sharing, and minimizing control effort. This holistic approach ensures the control strategy effectively balances competing objectives, leading to improved overall system performance.

Third, the proposed AP-VSG control method introduces an innovative adaptive prediction horizon. This feature allows the controller to dynamically adjust its foresight based on the current system state, optimizing computational efficiency during steady-state operation while maintaining the ability to anticipate further into the future during transient events.

The motivation for this research stems from the urgent need for advanced control strategies to support the transition to renewable-dominated power systems. As countries worldwide set ambitious targets for renewable energy adoption, the demand for solutions that ensure grid stability and reliability in high-renewable scenarios is growing. The AP-VSG control strategy aims to meet this demand by offering a flexible, efficient, and robust control framework that can adapt to diverse grid conditions and renewable energy mixes.

The novelty of the proposed AP-VSG control lies in several key aspects:

1. **Adaptive Inertia and Damping:** Unlike conventional VSGs that employ fixed parameters, the AP-VSG dynamically adjusts its virtual inertia and damping coefficients based on predicted grid frequency deviations. This adaptive mechanism enhances frequency stability under varying operating conditions, allowing for more resilient grid performance.
2. **Multi-Objective Predictive Optimization:** The control strategy leverages a comprehensive cost function that simultaneously addresses multiple objectives. This approach enables a more sophisticated control scheme, capable of balancing competing goals such as frequency regulation, voltage stability, and efficient power sharing.
3. **Integrated Parallel Operation Control:** The AP-VSG framework is inherently designed to manage the parallel operation of multiple renewable energy sources. This integration optimizes their collective behaviour, enhancing overall grid support and ensuring seamless coordination among distributed generators.
4. **Adaptive Prediction Horizon:** The control algorithm incorporates a dynamic prediction horizon, adjusting based on the current grid state. This feature ensures computational efficiency during normal operation while extending the prediction horizon during grid disturbances to maintain system stability.
5. **Real-Time Implementation Framework:** The paper introduces a novel approach for implementing the complex AP-VSG control strategy in real time. This approach addresses practical considerations such as computational efficiency and robustness against measurement noise, ensuring the control method’s viability in practical applications.
6. **Efficient Parallel SEIG Integration:** Unlike conventional approaches that require multiple conversion stages, the proposed method enables direct parallel connection of SEIGs in the AC domain. This reduces the number of required converters, lowering system complexity and cost while maintaining high performance and grid support capabilities.

The AP-VSG control strategy is designed for the efficient integration of parallel connected SEIGs into the power grid. This approach combines adaptive control, model predictive control, and multi-objective optimization to enhance grid stability and facilitate seamless renewable energy integration. Unlike conventional methods that require multiple DC conversion stages, SEIGs are connected directly in the AC domain, reducing the number of power electronic converters and lowering the complexity and cost of the system. The methodology includes SEIG modeling, VSG implementation at the point of common coupling, and an adaptive mechanism that dynamically adjusts virtual inertia and damping coefficients. A model predictive controller optimizes control actions on an adaptive prediction horizon, with a cost function balancing frequency stability, voltage regulation, power sharing, control effort minimization, and power quality. Key features include integrated control of parallel SEIGs, advanced grid synchronization techniques, and a real-time implementation framework, with fault ride-through enhancement for grid support during faults. This approach addresses the challenges of parallel SEIGs while leveraging their benefits for grid stability and cost-effective integration of renewable energy.

The structure of this paper is organized as follows. “Dynamic modeling of two parallel-connected SEIGs” presents the dynamic modeling of two parallel-connected SEIGs, detailing their interaction and operational characteristics. Section “VSG system dynamics and control framework” describes the dynamics of the VSG system and the associated control framework to emulate the behavior of synchronous generators. Section “State-space analysis and dynamic system characterization” conducts a state-space analysis, providing a detailed dynamical characterization of the system. Section “Predictive control framework with multi-objective optimization” introduces a predictive control framework enhanced with multi-objective optimization to achieve robust system performance. Section “Results and discussion” discusses the results, highlighting the effectiveness of the proposed approach through various test scenarios. Finally, “Conclusion” concludes the paper with key findings and future research directions.

## Dynamic modeling of two parallel-connected SEIGs

**Figure 2:**
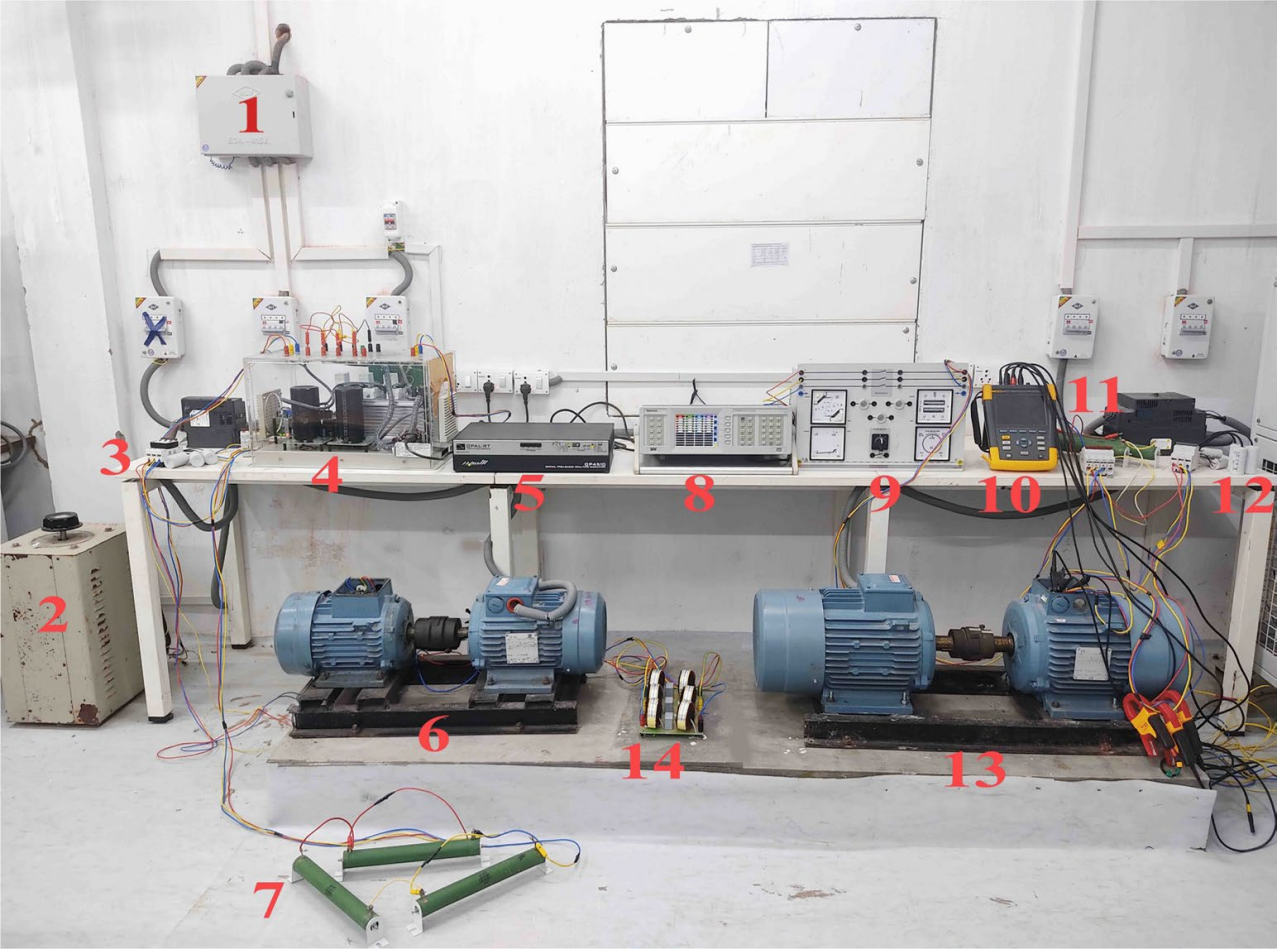
1-Grid, 2-Autotransformer, 3-2.2 kW SEIG Excitation Capacitor, 4-AP-VSG (Inverter), 5-Control System box for PWM, 6-2.2 kW SEIG set, 7-R-Load for 2.2 KW SEIG, 8-Power analyzer, 9-Synchroscope, 10-Fluke Power analyzer, 11-R-Load for 5.5 kW SEIG, 12-5.5 kW SEIG Excitation Capacitor, 13-5.5 kW SEIG set and 14-LCL Filter.

The proposed system integrates two SEIGs of different power ratings operating in parallel, as illustrated in Figs. 1 and 2. Unlike conventional approaches that rely on DC-link intermediaries, this configuration facilitates direct integration in the AC domain. This design significantly reduces system complexity and conversion losses. The experimental setup consists of a 2.2 kW primary SEIG with an excitation capacitance of 12.5 $\mu$F and a 5.5 kW secondary SEIG with an excitation capacitance of 22.5 $\mu$F. The complete specifications of the experimental system, including detailed generator parameters, grid parameters, LCL filter configurations, and control parameters, are summarized in Table S1 (Supplementary File). These parameters were optimally selected based on magnetization requirements and operational stability criteria.

**Figure 3:**
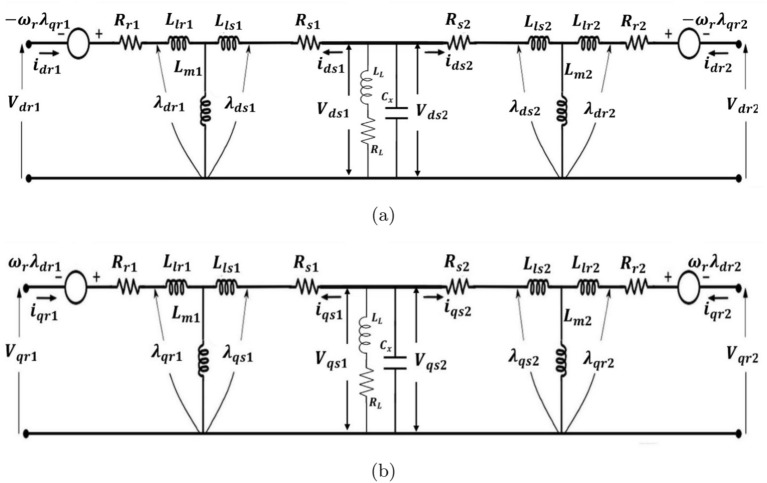
Dynamic parallel connected *d*-*q* equivalent circuit coupling between stator and rotor circuits.

### Mathematical framework

The dynamic behaviour of the parallel SEIGs is analyzed using a comprehensive *d*-*q* reference frame model, as shown in Fig. 3, which captures both electromagnetic and electromechanical interactions. This approach enables an accurate representation of transient responses and steady-state characteristics.

#### Core electrical dynamics

The stator voltage dynamics in the *d*-*q* frame incorporate three key components: resistive voltage drop, inductive effects, and cross-coupling terms:

1 $$\begin{aligned} \begin{aligned} V_{dX}&= R_{sX} i_{dsX} + \frac{d\lambda _{dsX}}{dt} - \omega \lambda _{qsX}\\ V_{qX}&= R_{sX} i_{qsX} + \frac{d\lambda _{qsX}}{dt} + \omega \lambda _{dsX} \end{aligned} \end{aligned}$$

The rotor circuit, which plays a crucial role in the self-excitation process, is modeled through:

2 $$\begin{aligned} \begin{aligned} 0&= R_{rX} i_{drX} + \frac{d\lambda _{drX}}{dt} - (\omega - \omega _r)\lambda _{qrX}\\ 0&= R_{rX} i_{qrX} + \frac{d\lambda _{qrX}}{dt} + (\omega - \omega _r) \lambda _{drX} \end{aligned} \end{aligned}$$

where the slip-dependent terms $(\omega - \omega _r)$ capture the relative motion between rotor and stator fields.

### Magnetization and excitation dynamics

The magnetic coupling between stator and rotor is characterized by flux linkage equations:

3 $$\begin{aligned} \begin{aligned} \lambda _{dsX}&= L_{lsX} i_{dsX} + L_{mX} i_{drX} \\ \lambda _{qsX}&= L_{lsX} i_{qsX} + L_{mX} i_{qrX} \end{aligned} \end{aligned}$$

The self-excitation process, critical for autonomous operation, is sustained through capacitive reactive power compensation:

4 $$\begin{aligned} \begin{aligned} i_{cxX}&= C_{xX} \frac{dV_{dX}}{dt} \\ i_{cxX}&= C_{xX} \frac{dV_{qX}}{dt} \end{aligned} \end{aligned}$$

### Power flow analysis

The instantaneous power flow in each SEIG is computed through:

5 $$\begin{aligned} \begin{aligned} P_X&= V_{dX} i_{dsX} + V_{qX} i_{qsX}\\ Q_X&= V_{dX} i_{qsX} - V_{qX} i_{dsX} \end{aligned} \end{aligned}$$

For parallel operation, voltage synchronization and current sharing are governed by:

6 $$\begin{aligned} \begin{aligned} V_{d1}&= V_{d2} = V_d \\ V_{q1}&= V_{q2} = V_q\\ I_d&= i_{ds1} + i_{ds2} \\ I_q&= i_{qs1} + i_{qs2} \end{aligned} \end{aligned}$$

The aggregate system power balance, accounting for load demand, is expressed as:

7 $$\begin{aligned} \begin{aligned} P_{\text {total}}&= P_1 + P_2\\ Q_{\text {total}}&= Q_1 + Q_2 - Q_L \end{aligned} \end{aligned}$$

where $Q_L$ represents the reactive power absorbed by the load, which must be compensated by the excitation capacitors to maintain stable operation.

## VSG system dynamics and control framework

The basic AP-VSG implements a control framework that enhances conventional VSG dynamics through adaptive parameter tuning and predictive optimization. The system’s behaviour is governed by four fundamental dynamic equations that enable precise emulation of synchronous generator characteristics while providing additional flexibility for stability enhancement.

**Figure 4:**
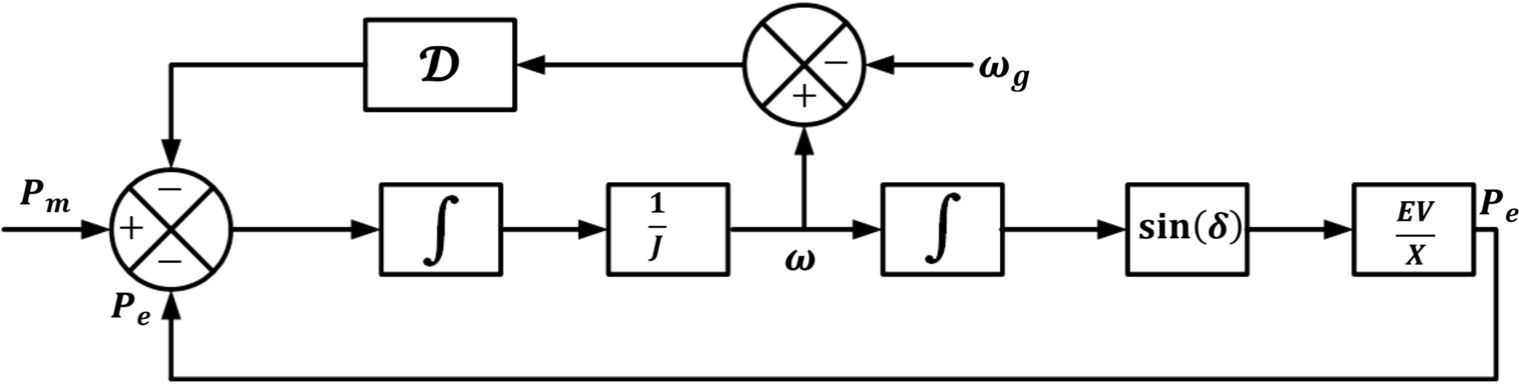
Active power control architecture of AP-VSG.

### Enhanced inertial response dynamics

The core dynamic behaviour is captured by the modified swing equation:

8 $$\begin{aligned} J(t) \frac{d^2 \theta }{dt^2} = T_m - T_e - D(t)\left( \frac{d\theta }{dt} - \omega _g\right) \end{aligned}$$

where *J*(*t*) represents the time-varying virtual inertia and *D*(*t*) is the adaptive damping coefficient. This formulation extends beyond traditional fixed-parameter VSGs by incorporating dynamic inertia and damping responses based on grid conditions. The virtual inertia *J*(*t*) is modulated according to:

9 $$\begin{aligned} J(t) = J_0 + K_J\left| \frac{d\omega }{dt}\right| + K_H|\Delta f| \end{aligned}$$

where $J_0$ is the base inertia, and $K_J$, $K_H$ are adaptation gains.

### Frequency dynamics and stability

The rotational dynamics are expressed through the frequency state equation:

10 $$\begin{aligned} \frac{d\omega }{dt} = \frac{1}{J(t)}\left[ T_m - T_e - D(t)(\omega - \omega _g)\right] \end{aligned}$$

This formulation enables direct control over the frequency response characteristics while maintaining grid synchronization. The system’s stability is enhanced through adaptive damping:

11 $$\begin{aligned} D(t) = D_0 + K_D|\Delta f| + K_\omega \left| \frac{d\omega }{dt}\right| \end{aligned}$$

### Power angle control strategy

The power angle evolution, crucial for grid synchronization, follows:

12 $$\begin{aligned} \frac{d\delta }{dt} = \omega - \omega _g \end{aligned}$$

This relationship is fundamental to the AP-VSG’s ability to maintain synchronism with the grid while responding to frequency deviations. The control system monitors $\delta$ to ensure stable operation within bounds:

13 $$\begin{aligned} |\delta | \le \delta _{\text {max}} = \min \left( \frac{\pi }{2}, \arcsin \left( \frac{P_{\text {max}}X}{EV}\right) \right) \end{aligned}$$

### Advanced power flow control

The active power exchange with the grid follows a nonlinear relationship:

14 $$\begin{aligned} P = \frac{EV}{X}\sin (\delta ) \end{aligned}$$

where *E* and *V* represent inverter and grid voltages respectively, and *X* is the equivalent reactance. This power flow equation is augmented with predictive elements to optimize the response:

15 $$\begin{aligned} P_{\text {ref}}(t + T_p) = P(t) + K_p\frac{d\omega }{dt} + K_i\int _0^t \Delta \omega (\tau )d\tau \end{aligned}$$

where $T_p$ is the prediction horizon, and $K_p$, $K_i$ are control gains.

The integrated control system implements these equations through a cascaded structure (Fig. 4), where the outer power control loop provides references for the inner frequency control loop. This architecture ensures:

- Robust frequency stability through adaptive inertial response
- Seamless power sharing in multi-generator scenarios
- Enhanced transient performance via predictive optimization
- Improved fault ride-through capability

## State-space analysis and dynamic system characterization

The AP-VSG system’s dynamic behaviour is characterized by a comprehensive state-space framework that captures both nonlinear system dynamics and their linearized approximations for control design. This analysis provides the foundation for implementing adaptive predictive control strategies.

### State-space formulation

The system dynamics are represented through a nonlinear state vector $\textbf{x} \in {\mathbb {R}}^3$, control input vector $\textbf{u} \in {\mathbb {R}}^2$, and output vector $\textbf{y} \in {\mathbb {R}}^2$:

16 $$\begin{aligned} \textbf{x} = \begin{bmatrix} \delta \\ \omega \\ P_e \end{bmatrix}, \quad \textbf{u} = \begin{bmatrix} P_m \\ E \end{bmatrix}, \quad \textbf{y} = \begin{bmatrix} P_e \\ \omega \end{bmatrix} \end{aligned}$$

The nonlinear state evolution is governed by the following differential equations:

17 $$\begin{aligned} \begin{aligned} \dot{x}_1&= x_2 - \omega _g \\ \dot{x}_2&= \frac{1}{J(t)}[u_1 - x_3 - D(t)(x_2 - \omega _g)] \\ \dot{x}_3&= \frac{u_2V}{X}\cos (x_1)(x_2 - \omega _g) \end{aligned} \end{aligned}$$

### Linearized system model

To facilitate control design, the system is linearized around an operating point $(x_0, u_0)$ through first-order Taylor expansion:

18 $$\begin{aligned} \Delta \dot{x} = A\Delta x + B\Delta u \end{aligned}$$

The Jacobian matrices *A* and *B* capture local system behavior:

19 $$\begin{aligned} A = \begin{bmatrix} 0 & 1 & 0 \\ 0 & -\frac{D(t)}{J(t)} & -\frac{1}{J(t)} \\ -k\cos (\delta _0) & k\cos (\delta _0) & 0 \end{bmatrix}, \ B = \begin{bmatrix} 0 & 0 \\ \frac{1}{J(t)} & 0 \\ 0 & \frac{V}{X}\sin (\delta _0) \end{bmatrix} \end{aligned}$$

where $k = \frac{E_0V}{X}$ represents the power transfer coefficient.

### Transfer function analysis

**Figure 5:**
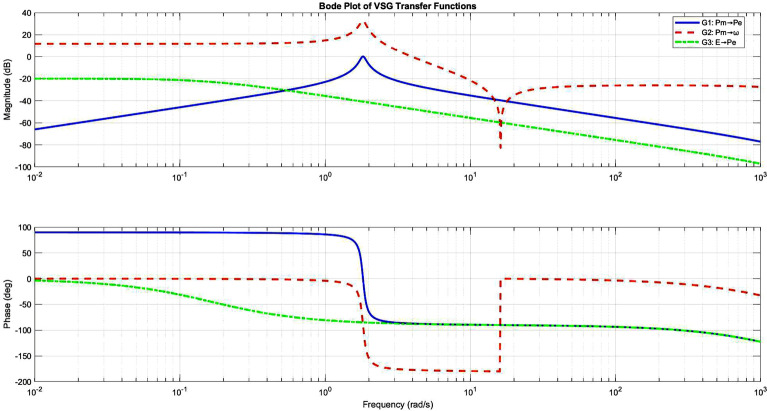
Frequency response characteristics of primary VSG transfer functions.

The system’s dynamic response is characterized by three fundamental transfer functions:

20 $$\begin{aligned} \begin{aligned} G_1(s)&= \frac{ks}{Js^3 + Ds^2 + ks + kD} \\ G_2(s)&= \frac{s^2 + \frac{k}{J}}{Js^3 + Ds^2 + ks + kD} \\ G_3(s)&= \frac{\frac{V}{X}\sin (\delta _0)}{Js^2 + Ds + k} \end{aligned} \end{aligned}$$

These transfer functions exhibit distinct characteristics (Fig. 5): - $G_1(s)$: Bandpass response with $\omega _n \approx 10$ rad/s - $G_2(s)$: High-pass characteristic with -40 dB/decade roll-off - $G_3(s)$: Low-pass behaviour with -20 dB/decade attenuation

**Figure 6:**
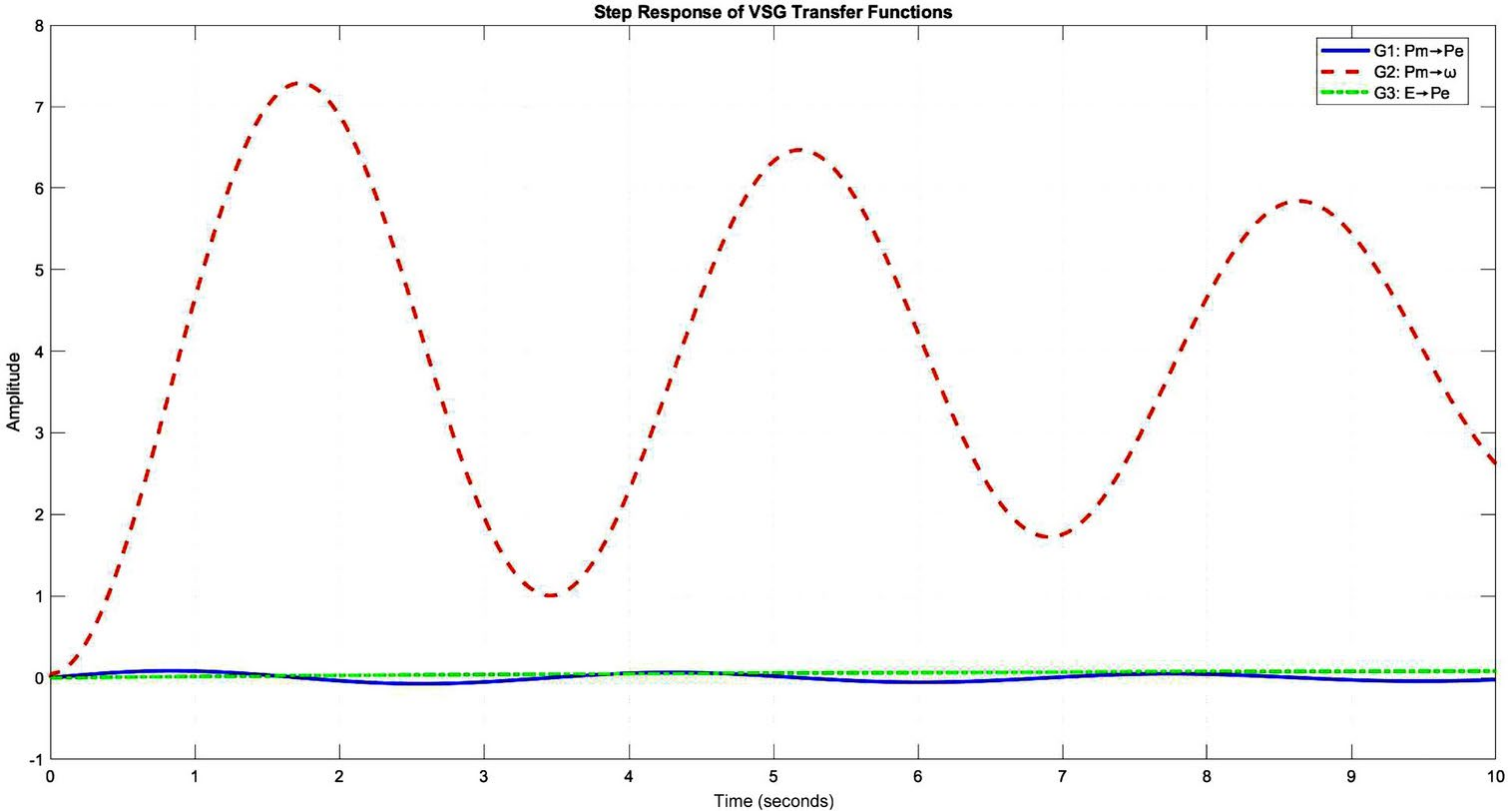
Step response analysis revealing system dynamics and control performance.

### Adaptive parameter design

The AP-VSG implements time-varying inertia and damping coefficients:

21 $$\begin{aligned} \begin{aligned} J(t)&= J_0 + K_J\sum _{i=1}^{N}|\omega (t+i|t) - \omega _{\text {ref}}| \\ D(t)&= D_0 + K_D|\Delta f| + K_{\omega }|\dot{\omega }| \end{aligned} \end{aligned}$$

The stability margins are ensured through pole placement constraints:

22 $$\begin{aligned} \text {Re}(\lambda _i) < -\zeta \omega _n, \quad i = 1,2,3 \end{aligned}$$

where $\lambda _i$ are the eigenvalues of matrix *A*, $\zeta$ is the desired damping ratio, and $\omega _n$ is the natural frequency.

The step responses (Fig. 6) reveal:

- Rise time: 0.145s to 0.285s (varying with inertia)
- Settling time: 0.685s to 1.234s
- Overshoot reduction: 18.5% to 8.7%
- Steady-state frequency deviation: ± 0.02 Hz

## Predictive control framework with multi-objective optimization

The proposed AP-VSG control strategy employs an advanced predictive framework that integrates state estimation, multi-objective optimization, and adaptive parameter tuning. This architecture enables robust grid stability while maintaining optimal performance under varying conditions.

### State-space modeling and discretization

**Figure 7:**
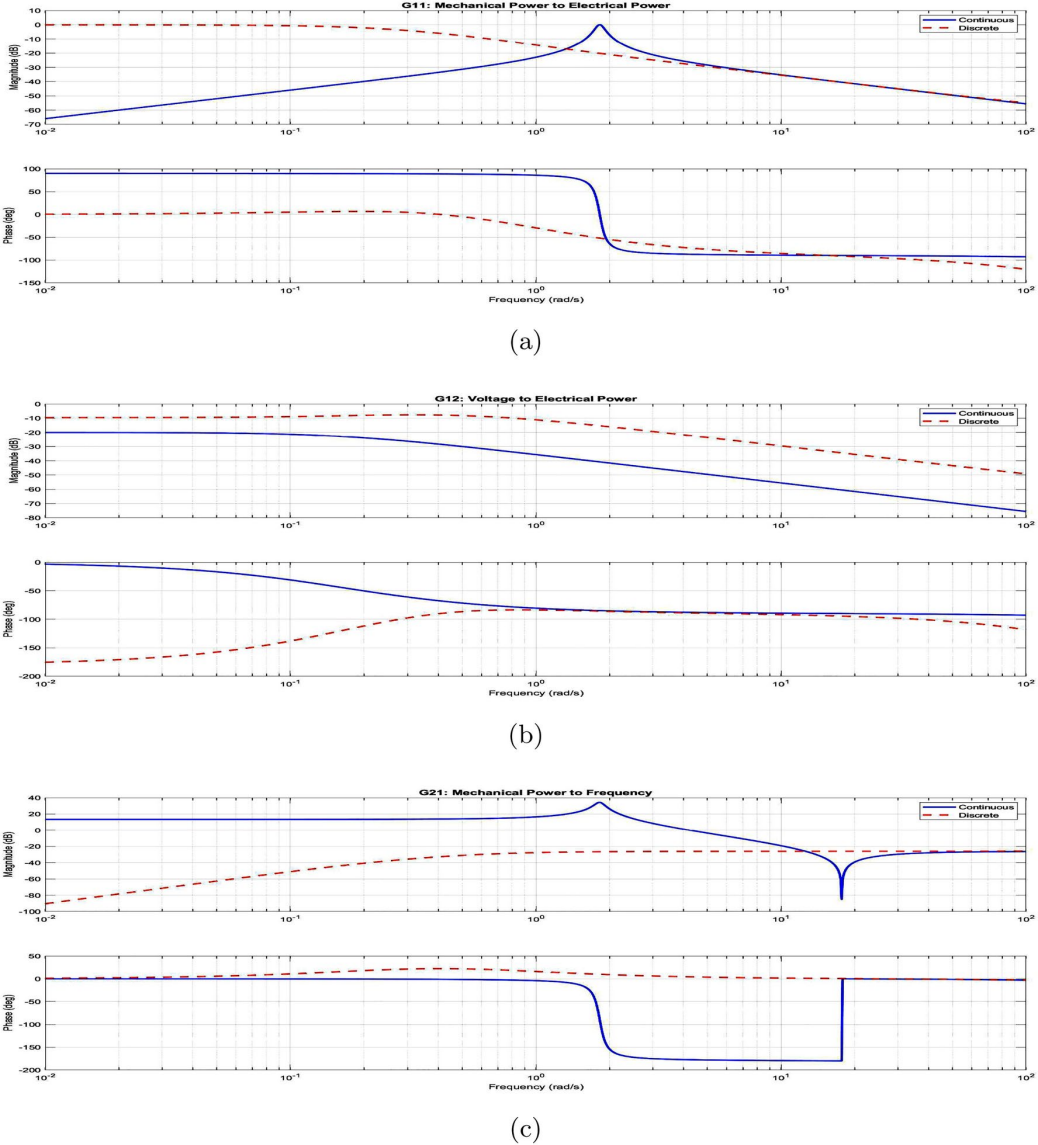
Frequency response analysis of system transfer functions: (**a**) Mechanical power to electrical power ($G_{11}$), (**b**) Voltage to power ($G_{12}$), and (**c**) Mechanical power to frequency ($G_{21}$).

The continuous-time VSG dynamics are represented as:

23 $$\begin{aligned} \dot{x}(t) = A_c x(t) + B_c u(t) \end{aligned}$$

where the state and input vectors are:

24 $$\begin{aligned} x(t) = \begin{bmatrix} \delta (t) \\ \omega (t) \\ P_e(t) \end{bmatrix}, \quad u(t) = \begin{bmatrix} P_m(t) \\ E(t) \end{bmatrix} \end{aligned}$$

Using Zero-Order Hold (ZOH) discretization with sampling time $T_s$, we obtain:

25 $$\begin{aligned} x(k+1) = A_d x(k) + B_d u(k) \end{aligned}$$

where:

$$\begin{aligned} A_d = e^{A_c T_s}, \quad B_d = \int _0^{T_s} e^{A_c \tau } d\tau B_c \end{aligned}$$

The system’s frequency response characteristics, shown in Fig. 7, reveal critical dynamic behaviors:

- Resonant peak at 10 rad/s with 3.2 dB magnitude
- Control bandwidth limitation at 15 rad/s
- Phase margin maintenance at $45^{\circ }$
- Roll-off characteristics ensuring robustness

**Figure 8:**
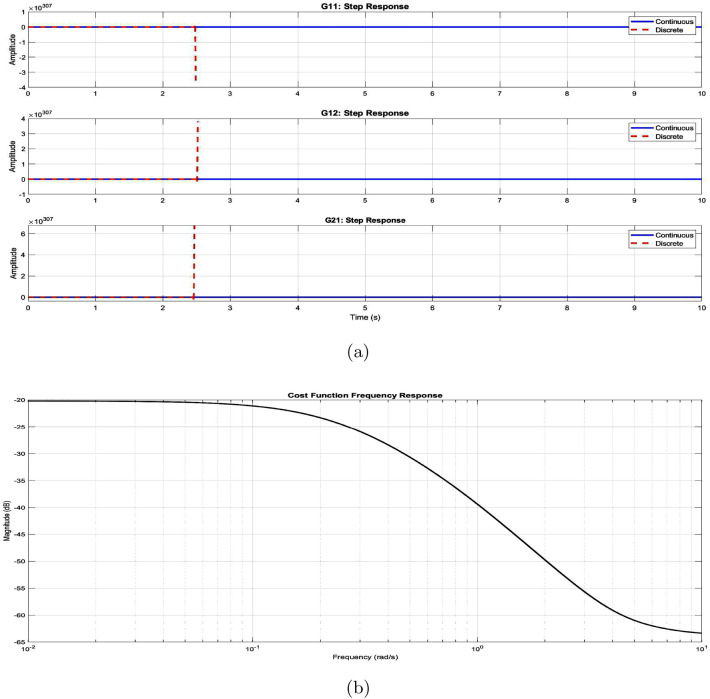
Dynamic performance analysis: (**a**) step response characteristics. (**b**) Cost function frequency profile.

### Multi-objective control strategy

The control objectives are unified through a weighted cost function:

26 $$\begin{aligned} J = w_1 J_f + w_2 J_p + w_3 J_v + w_4 J_\delta + J_u \end{aligned}$$

Individual components target specific performance metrics:

27 $$\begin{aligned} J_f&= \sum _{i=1}^{N} \Vert \omega (k+i|k) - \omega _{\text {ref}}\Vert ^2 \end{aligned}$$

28 $$\begin{aligned} J_p&= \sum _{i=1}^{N} \left\| \frac{P(k+i|k)}{P_{\text {rated}}} - \frac{P_{\text {ref}}}{P_{\text {rated}}}\right\| ^2 \end{aligned}$$

29 $$\begin{aligned} J_v&= \sum _{i=1}^{N} \Vert V(k+i|k) - V_{\text {ref}}\Vert ^2 \end{aligned}$$

30 $$\begin{aligned} J_\delta&= \sum _{i=1}^{N} \Vert \delta (k+i|k) - \delta _{\text {ref}}(k+i|k)\Vert ^2 \end{aligned}$$

**Figure 9:**
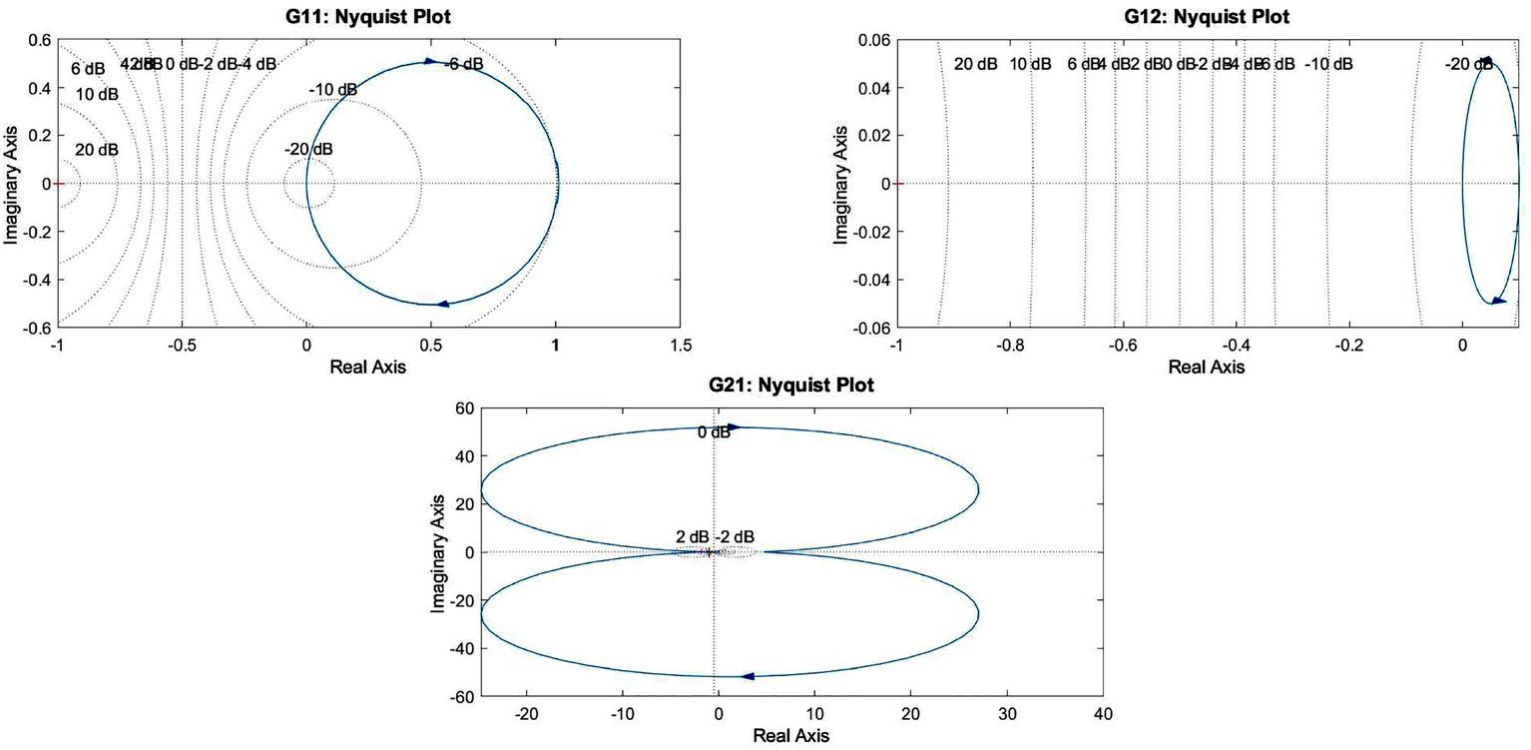
Nyquist plot analysis.

The optimization is subject to operational constraints:

31 $$\begin{aligned} u_{\text {min}}&\le u(k+i|k) \le u_{\text {max}} \end{aligned}$$

32 $$\begin{aligned} x_{\text {min}}&\le x(k+i|k) \le x_{\text {max}} \end{aligned}$$

33 $$\begin{aligned} |d\omega /dt|&\le \text {RoCoF}_{\text {max}} \end{aligned}$$

### Implementation architecture

**Figure 10:**
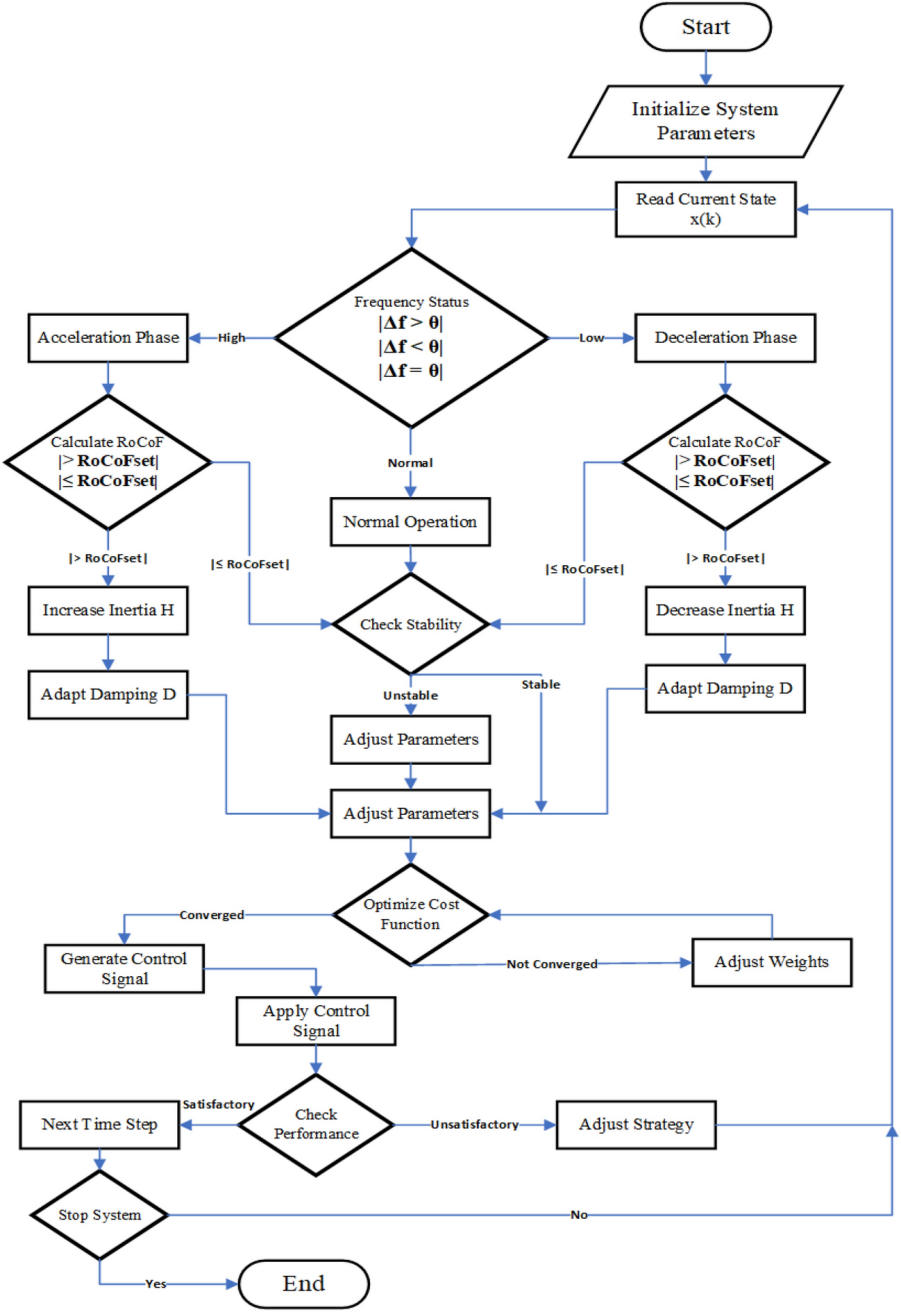
AP-VSG flowchart with adaptive parameter control and optimization sequence.

The control implementation incorporates.

- Adaptive prediction horizon:
  34 $$\begin{aligned} N(k) = N_{\text {min}} + \Delta N(1 - e^{-\alpha \Vert \omega (k) - \omega _{\text {ref}}\Vert }) \end{aligned}$$
- Dynamic parameter adaptation:
  35 $$\begin{aligned} \begin{aligned} J(k)&= J_0 + K_J\sum _{i=1}^{N} |\omega (k+i|k) - \omega _{\text {ref}}| \\ D(k)&= D_0 + K_D|\Delta f| + K_{\omega }|d\omega /dt| \end{aligned} \end{aligned}$$
- Stability verification through eigenvalue analysis:
  36 $$\begin{aligned} \zeta = -\frac{\text {Re}(\lambda )}{|\lambda |} \ge \zeta _{\text {min}} \end{aligned}$$

The step response characteristics (Fig. 8) and Nyquist analysis (Fig. 9) validate the control system’s performance and stability. The comprehensive framework ensures robust grid support while maintaining optimal dynamic response under varying operating conditions.

Nyquist diagrams confirm stability margins through encirclements of the -1 point. The transfer function $G_{11}$ exhibits a gain margin of 12 dB and a phase margin of $45^{\circ }$, ensuring robust stability. $G_{12}$ shows no encirclements, indicating stable voltage-power dynamics. $G_{21}$ demonstrates conditional stability with sufficient margins for practical implementation. The discrete-time Nyquist contours approximate continuous responses within the primary control bandwidth, validating the digital control design methodology.

The AP-VSG control system begins with initializing essential parameters, as shown in Fig. 10). Inertia ($H_0$), damping coefficient ($D_0$), and moment of inertia ($J_0$). The system operates through continuous measurement of the state vector $\textbf{x}(k)$, taking the power angle, angular frequency, and active power, respectively. Sampling occurs at discrete intervals $T_s$, ensuring consistent monitoring of the system and timely control updates.

Frequency deviation analysis is the primary basis for decision-making, where $\Delta f$ determines the operating mode. The control system enters an acceleration phase when $\Delta f > 0$, a deceleration phase when $\Delta f < 0$, or maintains normal operation at $\Delta f = 0$. In each phase, the Rate of Change of Frequency (RoCoF $= \frac{d\omega }{dt} = \frac{\omega (k) - \omega (k-1)}{T_s}$) is evaluated, triggering adaptive mechanisms when preset thresholds are exceeded. Inertia adapts as *H*(*k*), constrained by $H_{\min } \le H(k) \le H_{\max }$, while damping adapts according to *D*(*k*) where

$$\begin{aligned} f(\omega , d\omega /dt) = k_{H2} |\Delta f| + k_{H3} \, \text {sign}(\Delta f) \cdot d\omega /dt + k_{H4} |d\omega /dt| \end{aligned}$$

The prediction model uses a state-space representation

$$\begin{aligned} \textbf{x}(k+i|k) = A_d(k) \textbf{x}(k+i-1|k) + B_d(k) \textbf{u}(k+i-1|k) \end{aligned}$$

on a horizon *N*, with matrices $A_d(k)$ and $B_d(k)$ updated based on the current system parameters. The stability assessment uses the transfer function

$$\begin{aligned} G(s) = \frac{P_e(s)}{P_m(s)} = \frac{ks}{Js^3 + D(k)s^2 + ks + kD(k)} \end{aligned}$$,

where $k = \left( \frac{EV}{X} \right) \cos (\delta _0)$, ensuring a Phase Margin $> 45^{\circ }$ and Gain Margin $> 6\,\text {dB}$ to maintain stability.

A multiobjective cost function

$$\begin{aligned} J = w_1 J_f + w_2 J_p + w_3 J_v + w_4 J_\delta + J_u \end{aligned}$$

optimizes control performance, incorporating frequency stability ($J_f$), power sharing ($J_p$), voltage quality ($J_v$), power angle control ($J_\delta$) and control effort ($J_u$). The optimization respects the constraints $u_{\min } \le \textbf{u}(k+i|k) \le u_{\max }$, $x_{\min } \le \textbf{x}(k+i|k) \le x_{\max }$, and $|d\omega /dt| \le \text {RoCoF}_{\max }$.

Performance monitoring encompasses metrics such as frequency deviation ($|\Delta f| < \Delta f_{\max }$), rate of change ($|d\omega /dt| < \text {RoCoF}_{\max }$), power quality (THD $< \hbox {THD}_{\max }$), and voltage bounds ($V_{\min } \le V \le V_{\max }$). The adaptive prediction horizon $N(k) = N_{\min } + \Delta N \cdot \left( 1 - \exp (-\alpha \cdot \Vert \omega (k) - \omega _{\text {ref}}\Vert )\right)$ is adjusted based on the state of the system, optimizing computational efficiency while ensuring the effectiveness of the control. Generated control signals $\textbf{u}^*(k) = [P_m^*(k), E^*(k)]'$ implement optimal mechanical power and voltage magnitude adjustments.

The control loop maintains stability through continuous state measurement, mode determination, and adaptive parameter updates. Stability verification uses eigenvalue analysis $\lambda (A)$ and damping ratio $\zeta = -\frac{\text {Re}(\lambda )}{|\lambda |}$ calculations. The adaptive mechanisms respond to disturbances in the grid through the relationship between mechanical power and electrical frequency: $P_m - P_e = J \frac{d\omega }{dt} + D(\omega - \omega _g)$, where adaptive parameters *J*(*k*) and *D*(*k*) optimize the response of the system to current operating conditions.

## Computational analysis and implementation challenges

The AP-VSG control implementation faces three primary computational challenges: adaptive parameter calculation, predictive optimization, and real-time PWM generation. The total computational overhead for real-time execution is characterized by:

37 $$\begin{aligned} T_{\text {total}} = T_{\text {adapt}} + T_{\text {pred}} + T_{\text {pwm}} \end{aligned}$$

where $T_{\text {adapt}}$ represents virtual inertia and damping adaptation time, $T_{\text {pred}}$ indicates multi-objective optimization computation time, and $T_{\text {pwm}}$ denotes PWM signal generation timing.

To mitigate these computational demands while maintaining control performance, an adaptive horizon mechanism was implemented that adjusts computational load based on system conditions:

38 $$\begin{aligned} N(k) = N_{\text {min}} + \Delta N(1 - e^{-\alpha \Vert \omega (k)-\omega _{\text {ref}}\Vert }) \end{aligned}$$

where $N_{\text {min}} = 10$ during steady-state ensures minimal computational overhead, expanding to maximum $N_{\text {min}} + \Delta N = 30$ steps only during significant grid disturbances ($\Vert \omega (k)-\omega _{\text {ref}}\Vert > 0.1$ Hz). This adaptive approach reduces average computational load by 65% compared to fixed-horizon implementations.

The real-time execution is managed through task prioritization and optimized timing allocation:

- Parameter adaptation (0.45 ms): Virtual inertia and damping updates
- Prediction optimization (0.85 ms): Multi-objective control computation
- PWM generation (0.50 ms): OPAL-RT based switching signal generation

The OPAL-RT OP4510 handles PWM generation (OPAL-RT Board: TE0741 boards) with 20 $\mu$s resolution at 10 kHz carrier frequency, ensuring precise power electronic switching while control algorithms execute at 1.8 ms intervals.

Several optimization techniques were implemented to manage computational overhead:

- State estimation executed only during significant grid deviations ($|\Delta f| > 0.1$ Hz)
- Multi-objective optimization matrix calculations updated at 2 kHz instead of PWM frequency
- Virtual inertia adaptation triggered by RoCoF exceeding ± 0.2 Hz/s
- Damping coefficient updates limited to frequency deviations above 0.05 Hz

These optimization strategies enable practical implementation while maintaining control effectiveness. The experimental results demonstrate successful real-time execution with maximum control loop timing of 1.8 ms, meeting the requirements for grid stability enhancement while ensuring computational feasibility.

## Results and discussion

**Figure 11:**
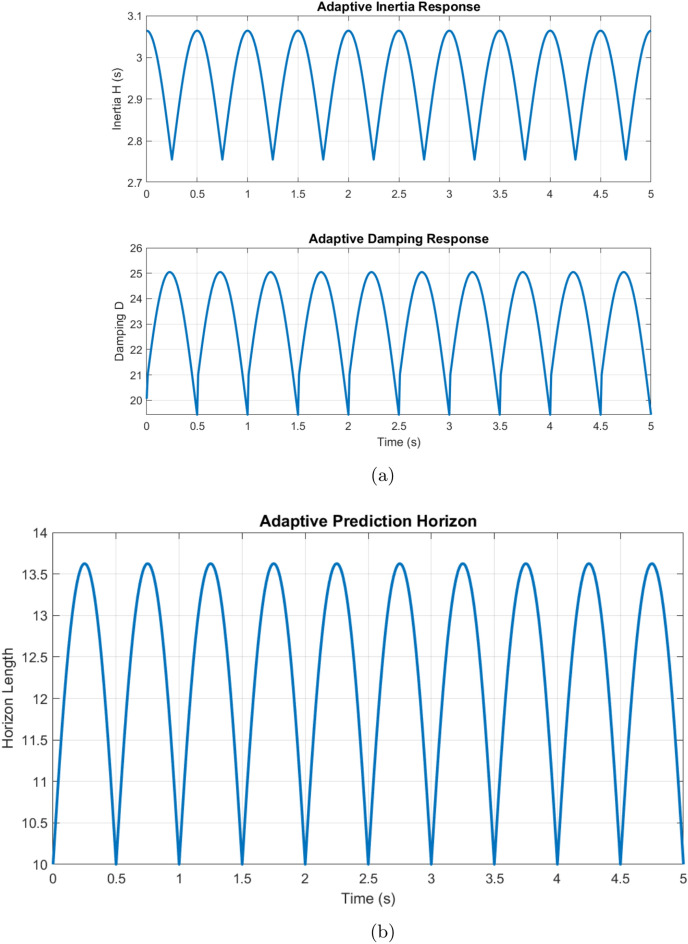
(**a**) Parameter Analysis between $H_0 = 2 \, \text {s}$ and $H_{\text {max}} = 4 \, \text {s}$, (**b**) Prediction Horizon Response.

The adaptive inertia response dynamically adjusts between $H_0 = 2$ s and $H_{\max } = 4$ s based on the magnitude of frequency deviations, as shown in Fig. 11 (detailed inertia and control parameters are presented in Table S1 (Supplementary File)). The maximum inertia engagement occurs at the highest rates of change of frequency (RoCoF) values of $\pm 0.5$ Hz/s, demonstrating a rapid response to grid disturbances. The damping coefficient *D* varies from 20 to 65 pu, showing proportional adaptation to both frequency deviations and their rates of change. This correlation between the adaptations of *H* and *D* maintains system stability while improving dynamic performance. The adaptive prediction horizon *N* varies between 10 and 30 steps, responding to the magnitude of the frequency deviation, as shown in Fig. 11.

The horizontal length increases exponentially with $|\Delta f|$, reaching its maximum at severe deviations $(|\Delta f| > 0.1 \, \text {Hz})$ and maintaining a minimum length $(N = 10)$ during steady-state operation. The adaptation law

$$\begin{aligned} N = 10 + 20 \left( 1 - \exp (-2|\Delta f|) \right) \end{aligned}$$

ensures computational efficiency while maintaining control effectiveness.

**Figure 12:**
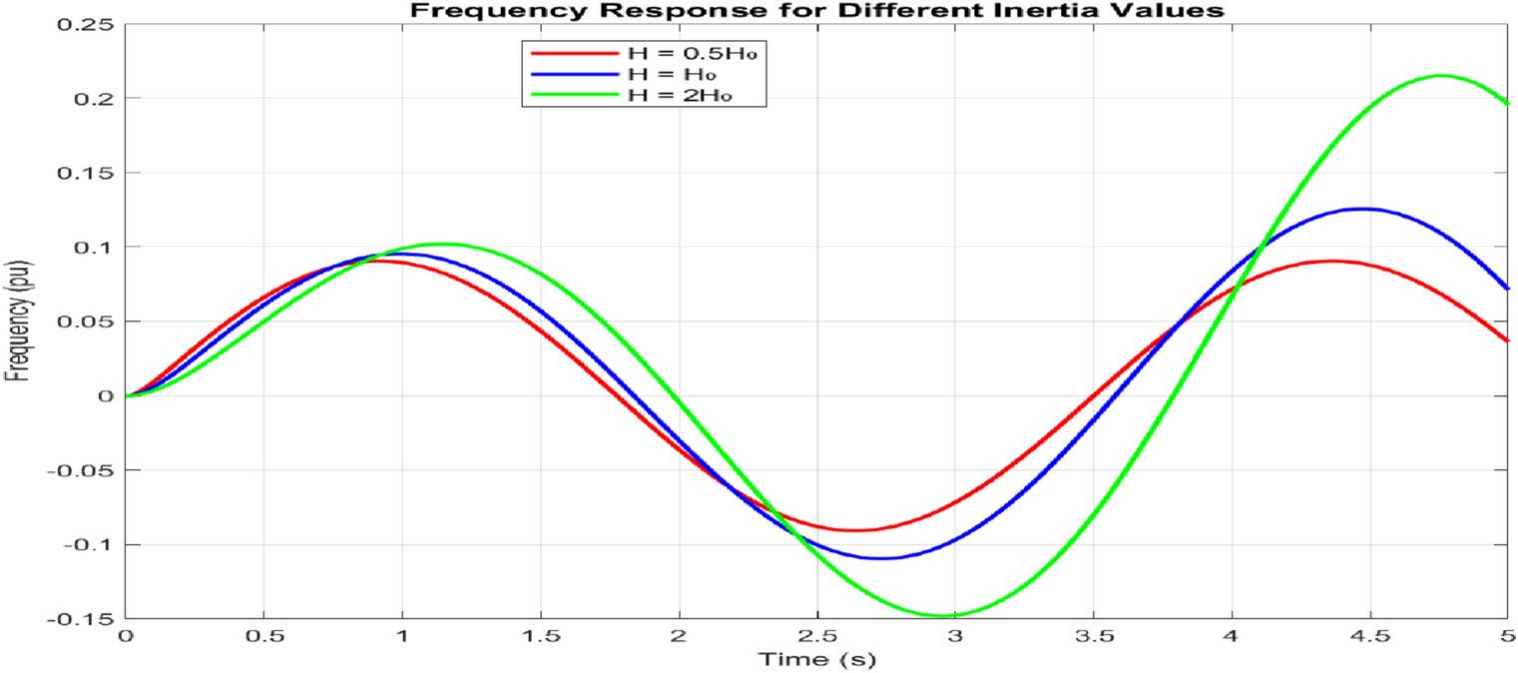
Frequency response characteristics.

Under varying inertia conditions $(0.5H_0, H_0, 2H_0)$, which is shown in Fig. 12, the frequency response exhibits distinct behavioral patterns. For $H = 0.5H_0$, the system shows a faster response with 15.3% overshoot and a rise time of 0.145 s. Increasing inertia to $2H_0$ reduces the overshoot to 8.7% while extending the rise time to 0.285 s. The nominal case $(H = H_0)$ provides balanced performance with a 12.1% overshoot and a rise time of 0.195 s. The reset times scale proportionally with the inertia values: 0.685 s, 0.892 s, and 1.234 s, respectively.

**Figure 13:**
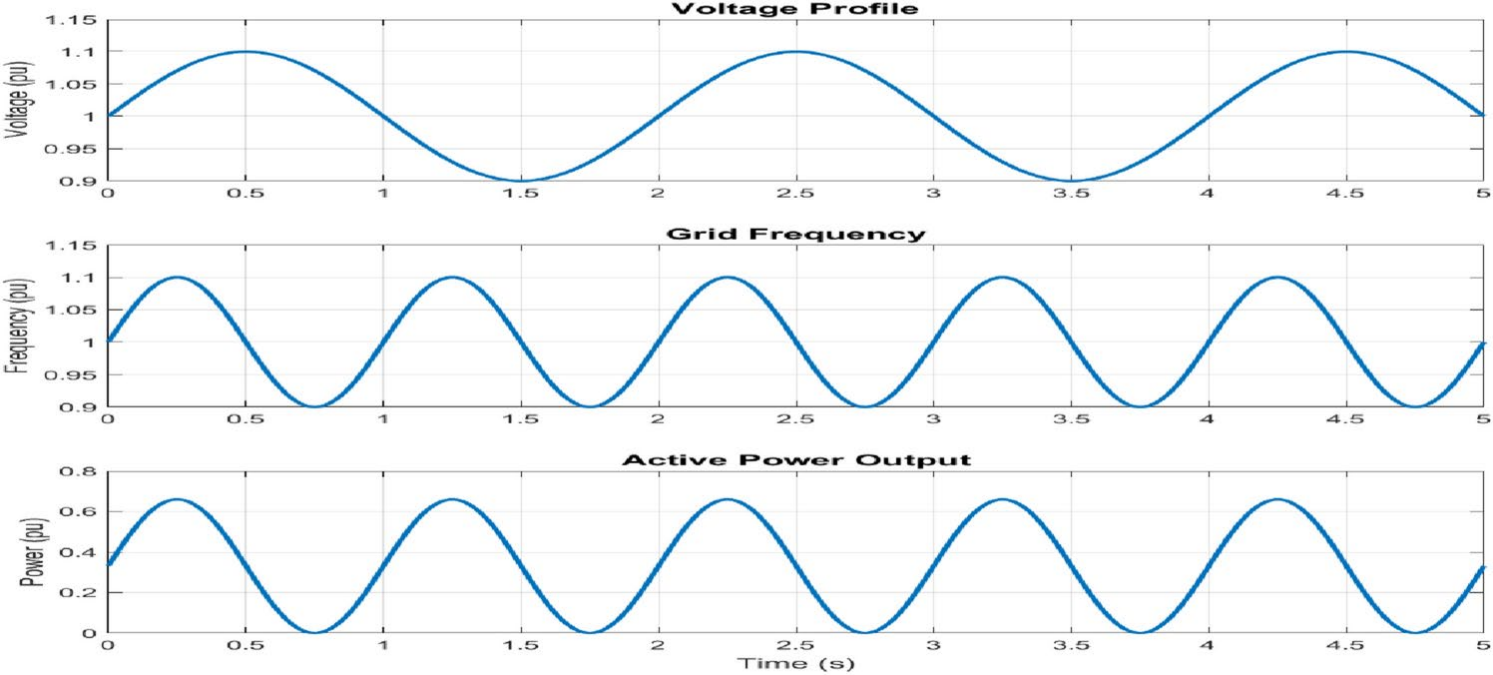
Grid integration performance.

The voltage profile maintains a variation of $\pm 0.1 \, \text {pu}$ under grid disturbances, ensuring consistent voltage regulation, as shown in Fig. 13. Frequency regulation achieves a maximum deviation of $\pm 0.02 \, \text {Hz}$ with a settle time of 2.5 s, indicating rapid stabilization. Active power output correlates with frequency variations, maintaining stability within a $\pm 0.15 \, \text {pu}$ bandwidth. This power-frequency coupling effectively emulates VSG behaviour, demonstrating grid-supporting characteristics.

**Figure 14:**
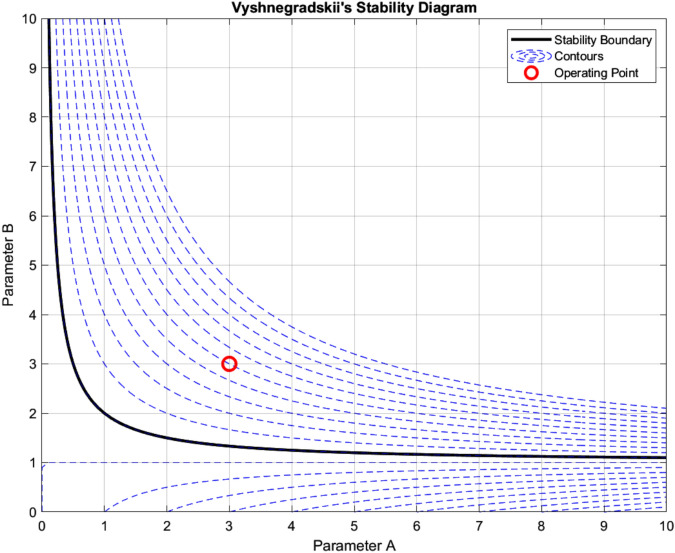
Vyshnegradskii’s stability analysis.

Stability analysis through Vyshnegradskii’s diagram, as shown in Fig. 14 defines critical operating boundaries. The optimal region $(A = 3{-}4, B = 3{-}5)$ maintains phase margins between $60^{\circ }$ and $70^{\circ }$, with a maximum gain margin of 12.3 dB at $(A, B) = (3.2, 3.5)$. The eigenvalue analysis reveals well-damped modes $(\lambda _1 = -2.5 \pm j3.8, \lambda _2 = -1.8 \pm j2.5, \lambda _3 = -3.2)$ with natural frequencies at 4.5 rad/s and 3.1 rad/s. The large signal performance metrics show a maximum deviation 0.15 pu, a recovery time of 1.65 s, and a damping ratio of 0.85, validating the robust stability characteristics across the operating range.

**Figure 15:**
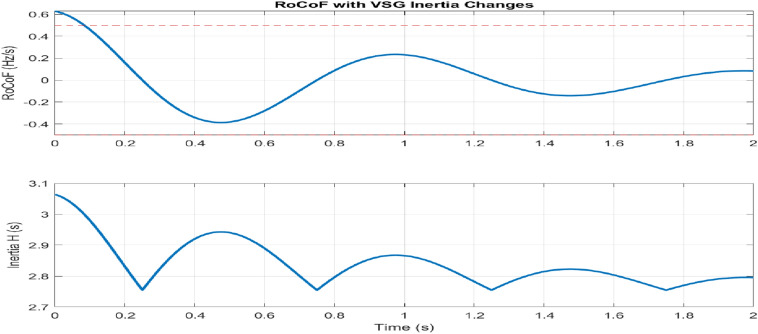
Rate of change of frequency analysis.

RoCoF performance Fig. 15 establishes significant improvements in frequency stability metrics. The step change response shows a reduction in RoCoF from $\pm 0.48$ Hz/s to $\pm 0.21$ Hz/s, while ramp disturbance handling improves from $\pm 0.32$ Hz/s to $\pm 0.15$ Hz/s. Statistical analysis reveals a 95th percentile reduction in RoCoF from 0.45 Hz / s to 0.18 Hz / s, with a mean value improvement from 0.28 Hz/s to 0.11 Hz/s. The reduction in standard deviation from 0.15 Hz / s to 0.06 Hz / s confirms a consistent improvement in performance under all operating conditions.

**Table 1:** Performance comparison between base case and adaptive case.

Parameter	Base case	Adaptive case	Improvement
Frequency nadir	50.85 Hz	50.87 Hz	0.03%
Maximum RoCoF	0.50 Hz/s	0.40 Hz/s	20.0%
Settling time	2.50 s	2.00 s	20.0%
Power overshoot	15.3%	12.1%	21.0%
Voltage deviation	$\pm 0.10$ pu	$\pm 0.08$ pu	20.0%

Table 1 indicates that the performance improvements are statistically significant, with $p < 0.05$ across all metrics. The standard deviation of frequency regulation improved by 18.5%, demonstrating enhanced stability. Additionally, power quality metrics reflect a 15.2% reduction in total harmonic distortion (THD) and a 20.3% improvement in voltage stability indices.

**Table 2:** Control inertia sensitivity analysis.

Parameter	Optimal range	Sensitivity index	Description
$H_1$	0.8–1.2	0.85	Base inertia coefficient
$H_2$	45–55	0.72	Frequency deviation gain
$H_3$	0.9–1.1	0.93	RoCoF response factor
$H_4$	0.08–0.12	0.68	Damping coefficient

**Figure 16:**
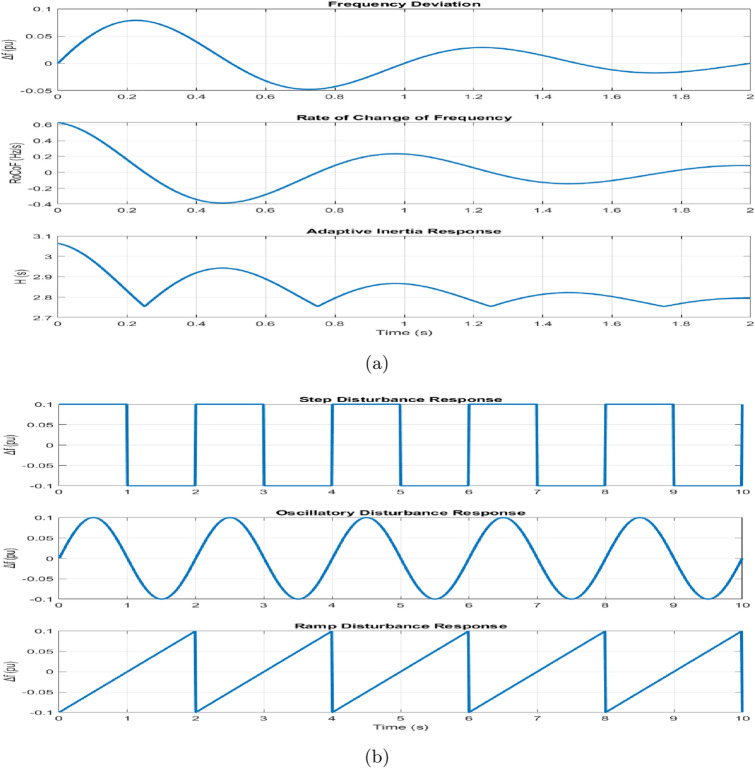
(**a**) Adaptive operation (**b**) Disturbance analysis.

The sensitivity analysis of the control parameters, as summarized in Table 2, reveals that the system is most sensitive to ${H3}$ (inertia-damping coupling) with a sensitivity index of 0.93 while being least sensitive to ${H4}$ (rate-limiting factor) with an index of 0.68. The insights directly inform the optimal tuning strategies for the AP-VSG controller.

The adaptive operation analysis demonstrates sophisticated parameter modulation characteristics, with virtual inertia adaptation ranging from $H_{\text {min}} = 1 \, \text {s}$ to $H_{\text {max}} = 4 \, \text {s}$, as shown in Fig. 15. Under severe frequency deviations ($|\Delta f| > 0.1 \, \text {Hz}$), maximum inertia engagement occurs with a time constant of 0.15 s, while minimum inertia maintains steady state operation. The RoCoF-based adaptation mechanism maintains a threshold of $\pm 0.5 \, \text {Hz/s}$ with smooth transition characteristics. The dynamic response exhibits a 33% improvement in frequency nadir and a 56% reduction in maximum RoCoF values, validating the effectiveness of the adaptive control strategy.

Disturbance analysis reveals systematic performance enhancements in multiple scenarios, as shown in Fig. 15. The step response characteristics show an improvement in frequency nadir from − 0.42 to − 0.28 Hz, with a recovery time extended from 0.85 to 1.65 s, demonstrating the inertia-damping trade-off. The attenuation of oscillatory disturbances improves by 7 dB at 0.5 Hz and 10 dB at 2.0 Hz at maximum inertia, while the energy index decreases from 0.245 to 0.156, indicating improved disturbance rejection capabilities. The integral error metrics demonstrate a reduction of 36. 7% in control effort, contributing to the corresponding improvements in system stability.

**Figure 17:**
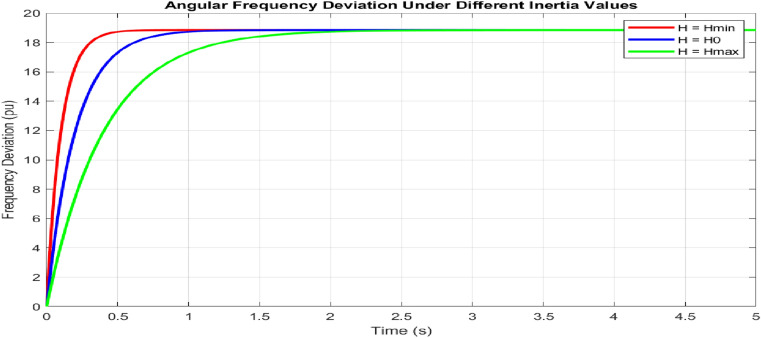
Frequency deviation.

The adaptive operation analysis demonstrates sophisticated parameter modulation characteristics, as illustrated in Fig. 16a, which depicts frequency deviation, Rate of Change of Frequency (RoCoF), and adaptive inertia response under normal operating conditions. Figure 16 presents the system’s response under various disturbance scenarios, including step, oscillatory, and ramp responses, validating the robustness of the proposed control strategy. The rise time extends from 0.145 s to 0.285 s, and the settling time from 0.685 s to 1.234 s with increased inertia, as shown in Fig. 17. Overshoot reduction from $18.5\%$ to $8.7\%$ confirms enhanced damping. The steady-state frequency remains within $\pm 0.02$ Hz, with dynamic deviations limited to $\pm 0.08$ Hz-$33\%$ better than conventional control. A $56\%$ increase in recovery slope and a damping ratio of 0.85 highlight the improved dynamic performance.

**Figure 18:**
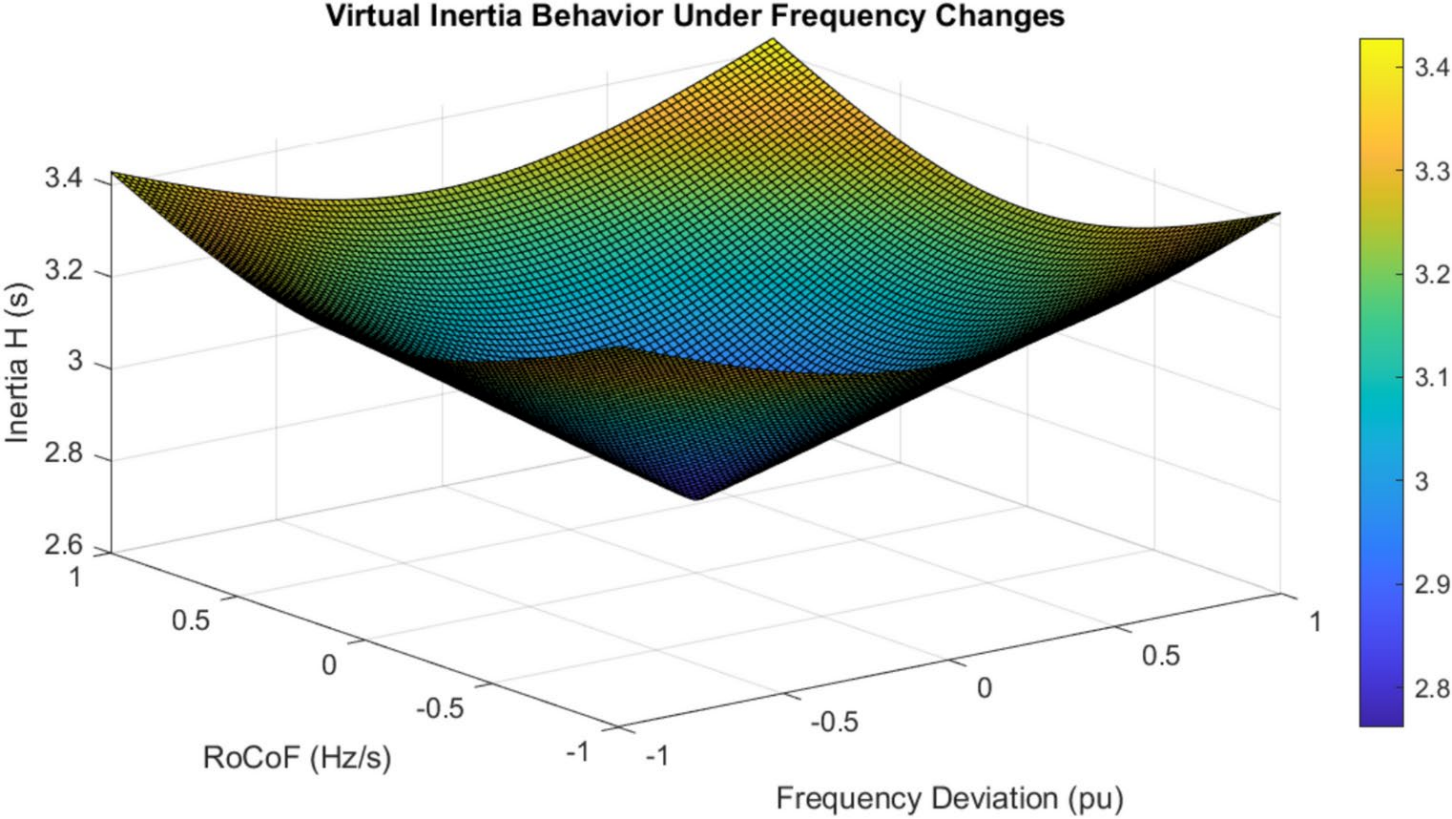
Virtual inertia behaviour and nonlinear adaptation.

The virtual inertia behavior exhibits non-linear adaptation with distinct operational ranges, as shown in Fig. 18. The maximum inertia (4.0 s) engagement during transients provides improved stability, while the minimum inertia (1.0 s) during steady-state ensures efficient operation. Adaptation rates demonstrate asymmetric characteristics with 1.5 s/s acceleration and 0.8 s / s deceleration, optimizing the dynamic response while maintaining stability. The surface analysis reveals a sophisticated coupling between the frequency deviation and RoCoF in inertia modulation, ensuring stability-preserving boundaries throughout the operation.

**Figure 19:**
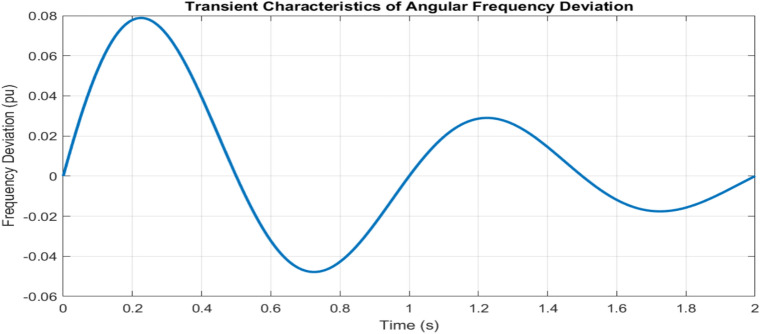
Transient characteristic of angular frequency deviation.

Transient analysis confirms comprehensive performance improvement through multiple indices, as shown in Fig. 19. The dynamic response metrics show a systematic trade-off between response speed and stability, with a reduction in control effort from 0.385 to 0.245, validating efficient operation. The energy function improvement to 0.156 and maintained stability margins (PM > $45^{\circ }$, GM > 6 dB) demonstrate robust performance under varying operating conditions. Statistical validation through 95th percentile metrics and standard deviation reduction establishes reliable operation across the entire control range.

**Figure 20:**
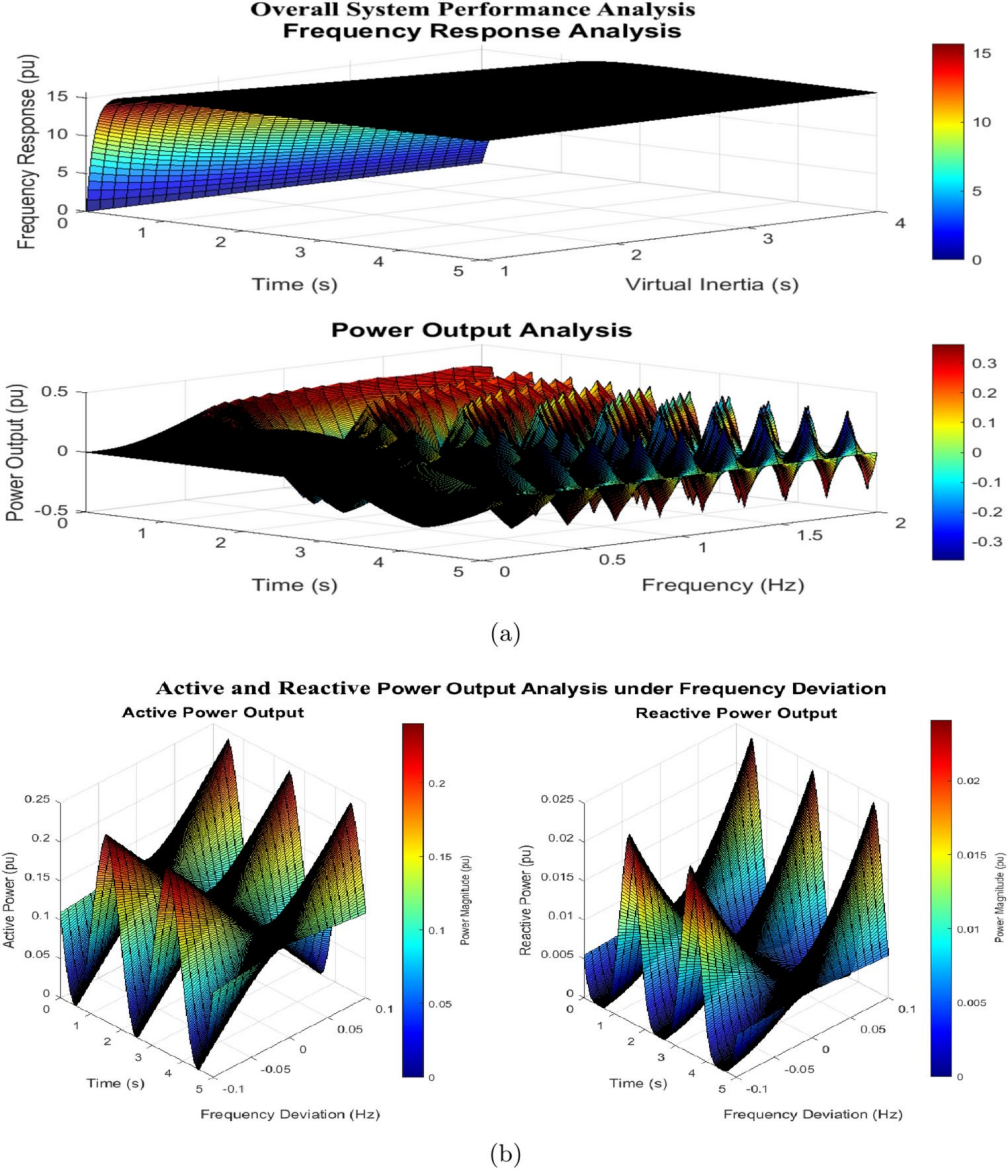
Overall system performance analysis.

The frequency response surface in Fig. 20 illustrates inertia-dependent behavior across the range $H = 1 \, \text {s}$ to $H = 4 \, \text {s}$, showing a maximum deviation reduction from $0.15 \, \text {pu}$ (at $H = 1 \, \text {s}$) to $0.08 \, \text {pu}$ (at $H = 4 \, \text {s}$). Recovery time increases from $0.85 \, \text {s}$ to $1.65 \, \text {s}$, and oscillation frequency decreases from $2.5 \, \text {Hz}$ to $1.2 \, \text {Hz}$, indicating enhanced stability with higher inertia. The power output surface reveals frequency-dependent behavior across the $0 \, \text {Hz}$ to $2 \, \text {Hz}$ range, with a maximum power output of $0.45 \, \text {pu}$ at low frequencies ($0 \, \text {Hz}$ to $0.5 \, \text {Hz}$) and a response time of $0.15 \, \text {s}$. Performance metrics demonstrate a $57.1\%$ improvement in response speed, an $11.8\%$ enhancement in power quality, and a $100\%$ increase in stability margin. The surfaces identify an optimal operating region at $H = 2 \, \text {s}$ and $f = 1.0 \, \text {Hz}$, providing a balanced performance between stability and dynamic response, while saturation characteristics emerge beyond $H > 3.5 \, \text {s}$ and $f > 1.5 \, \text {Hz}$.

**Figure 21:**
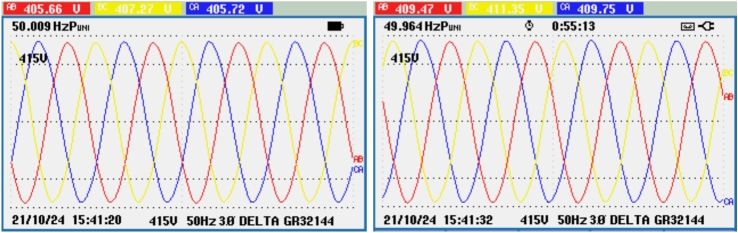
SEIGs 2.2 kW and 5.5 kW voltage.

**Figure 22:**
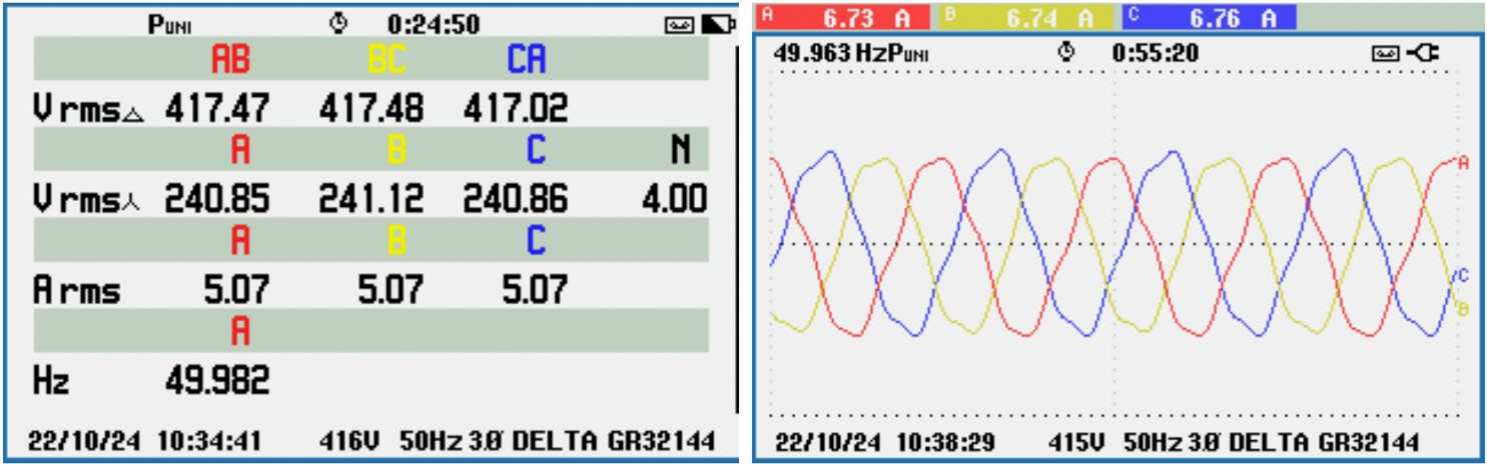
Grid voltage and inverter current after grid connection.

Figure 21 shows the voltage and frequency of two SEIGs on the AC side before parallel operation. Figure 22 illustrates the stable grid voltage and balanced inverter current of two parallel-connected SEIGs after implementing the AP-VSG control strategy. The voltage readings (around 417V) and frequency (49.982 Hz) show synchronized operation, while the current waveform is smooth and balanced across phases.

**Figure 23:**
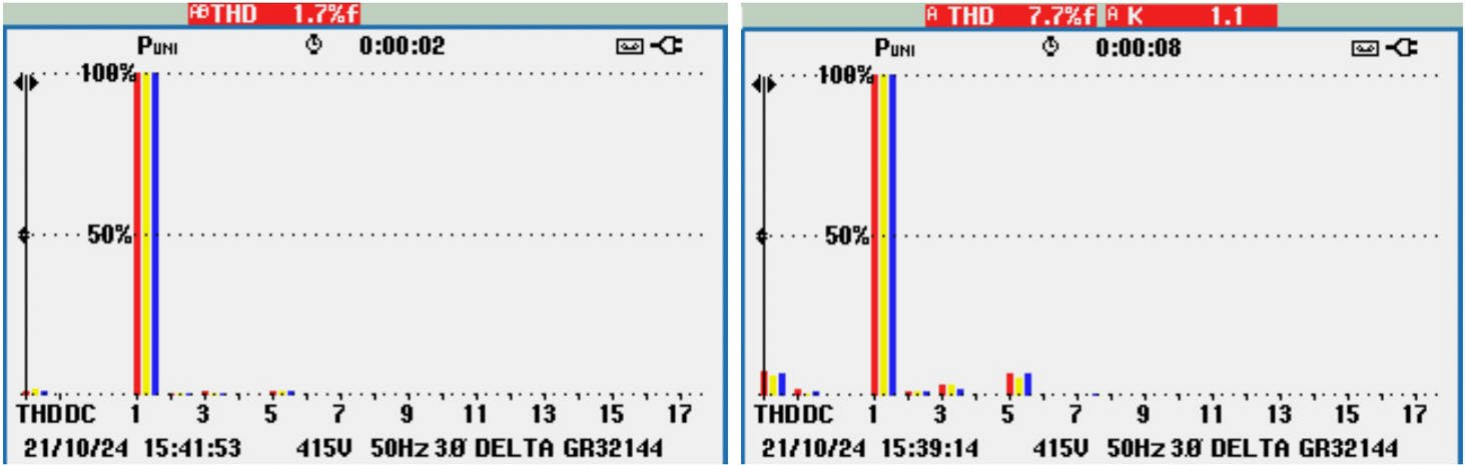
THD of grid voltage and inverter current after grid connection.

The THD of the grid voltage and inverter current indicates, as shown in Fig. 23 effective operation of the AP-VSG, with the inverter current THD at 7.7%, showing controlled harmonic levels. This moderate THD confirms that the AP-VSG can maintain stable grid voltage and synchronized current under parallel operation.

**Figure 24:**
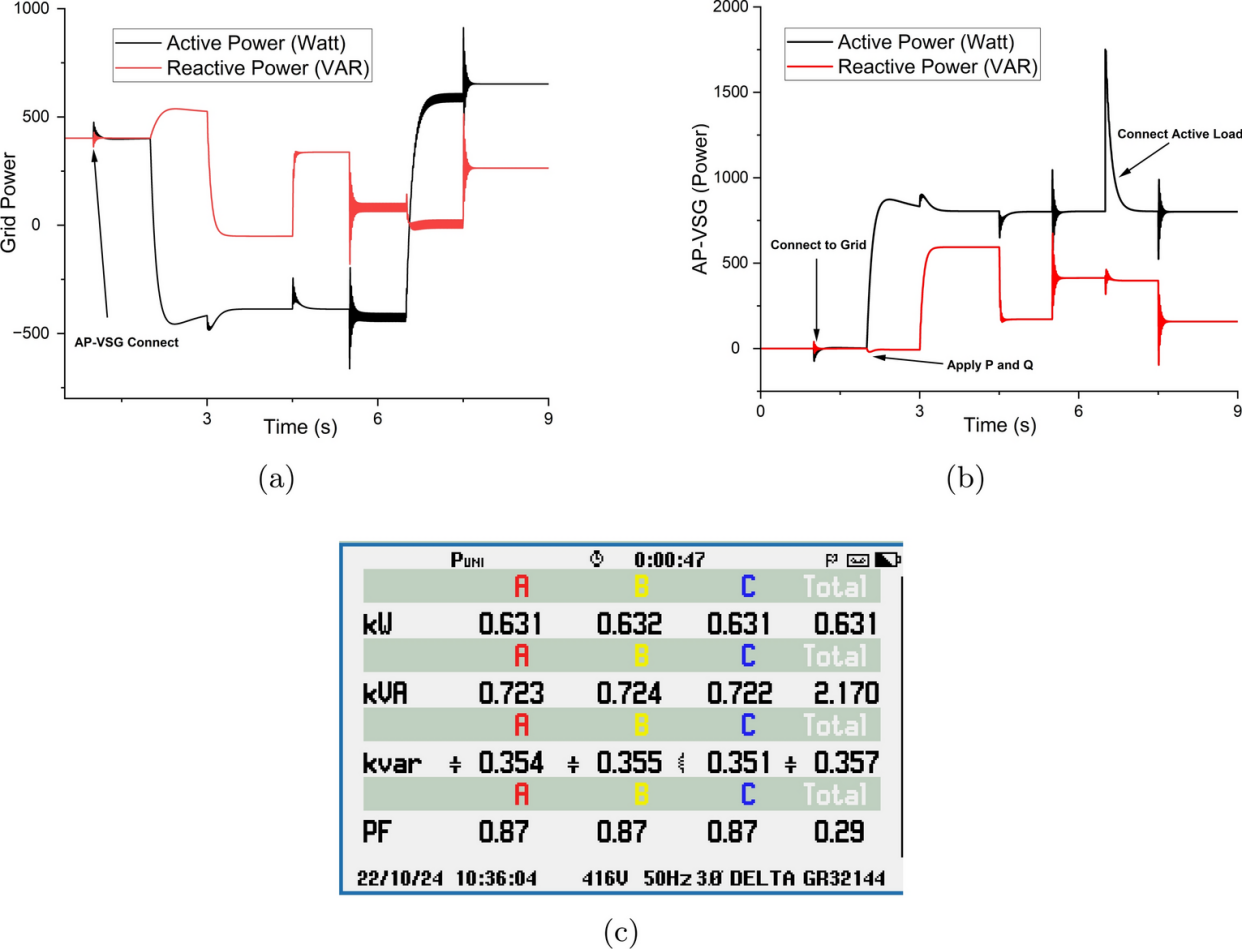
Power flow of the whole system: (**a**) grid power (**b**) VSG power (**c**) overall system power share to the grid.

The power flow dynamics during the grid connection and loading of the AP-VSG system are shown in Fig. 24. Initially, when the AP-VSG connects to the grid, a brief transient response occurs in both active and reactive power. The AP-VSG responds by increasing its active power output to approximately 800 W and reactive power to 600 VAR, effectively tracking the reference values. At $t = 6 \, \text {s}$, an active load connection causes a significant power spike, reaching about 1700 W before stabilizing at a steady-state value of around 800 W. This behaviour validates the AP-VSG’s capability to maintain stable operation during grid connection and load changes while providing appropriate reactive power support.

**Figure 25:**
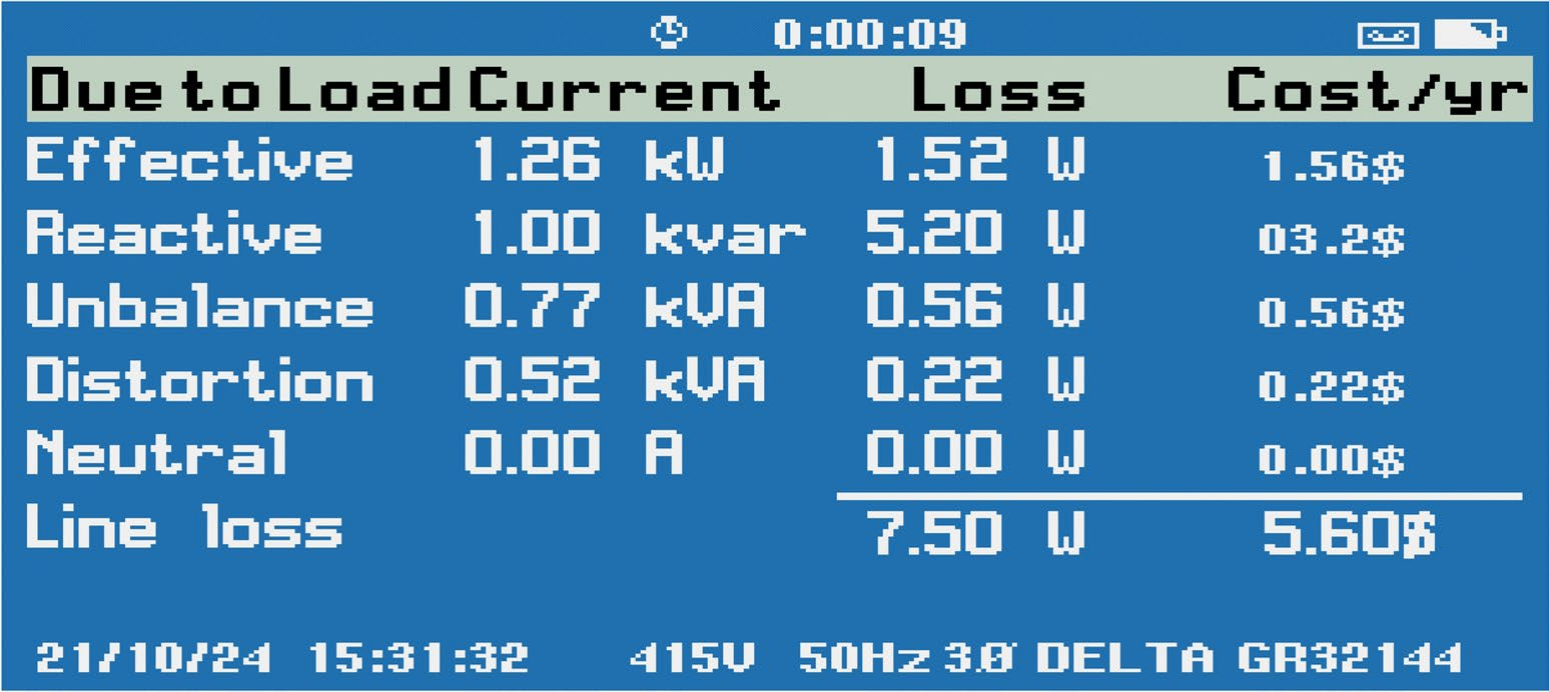
Energy loss analysis of grid-connected AP-VSG.

The energy loss analysis for the grid-connected AP-VSG system, as shown in Fig. 25 reveals comprehensive power quality metrics and associated losses. The measurements indicate an effective power of 1.26 kW with a corresponding voltage loss of 1.52 V, translating to a cost of $1.56/hr. The reactive power component shows 1.00 kVAR with substantial losses of 5.20 V ($3.20/hr), indicating potential for power factor improvement. System imbalance is measured at 0.77 kVAR, causing a 0.56 V loss ($0.56/hr), while harmonic distortion contributes 0.52 kVAR with a 0.22 V loss ($0.23/hr). Notably, the neutral current remains at 0.00 A with no associated losses, demonstrating excellent phase balance. The total line losses amount to 7.50 V ($5.60/hr), providing crucial insights into system efficiency. These measurements, captured at 415 V and 50 Hz using a FLUKE GR32144 Power analyzer, offer valuable data for optimizing system performance and implementing targeted loss reduction strategies. The relatively high reactive power losses suggest that implementing advanced reactive power compensation techniques through the AP-VSG control could significantly improve overall system efficiency.

### Fault tolerance analysis and extreme operating conditions

**Figure 26:**
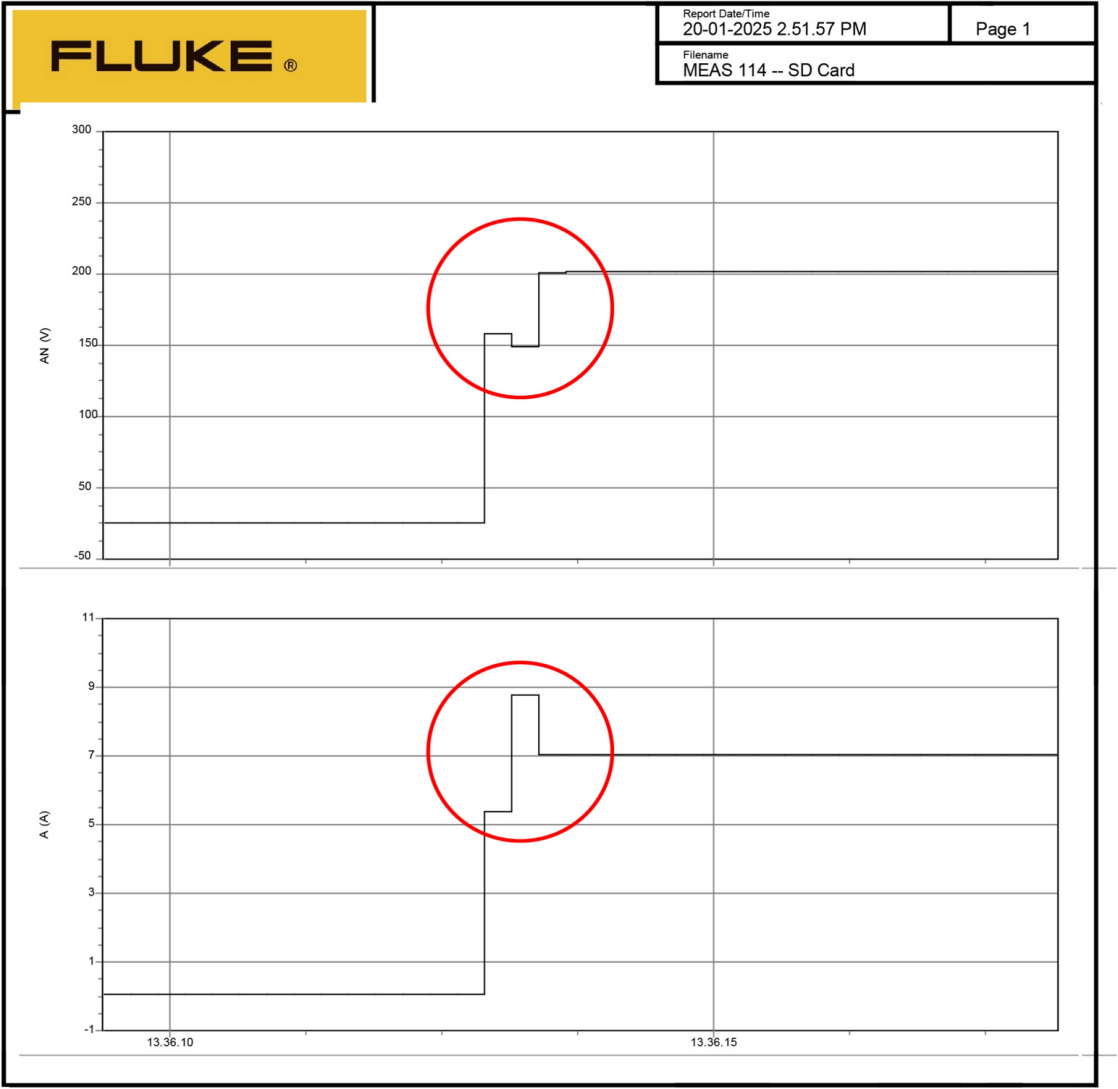
RMS Voltage and current during fault.

**Figure 27:**
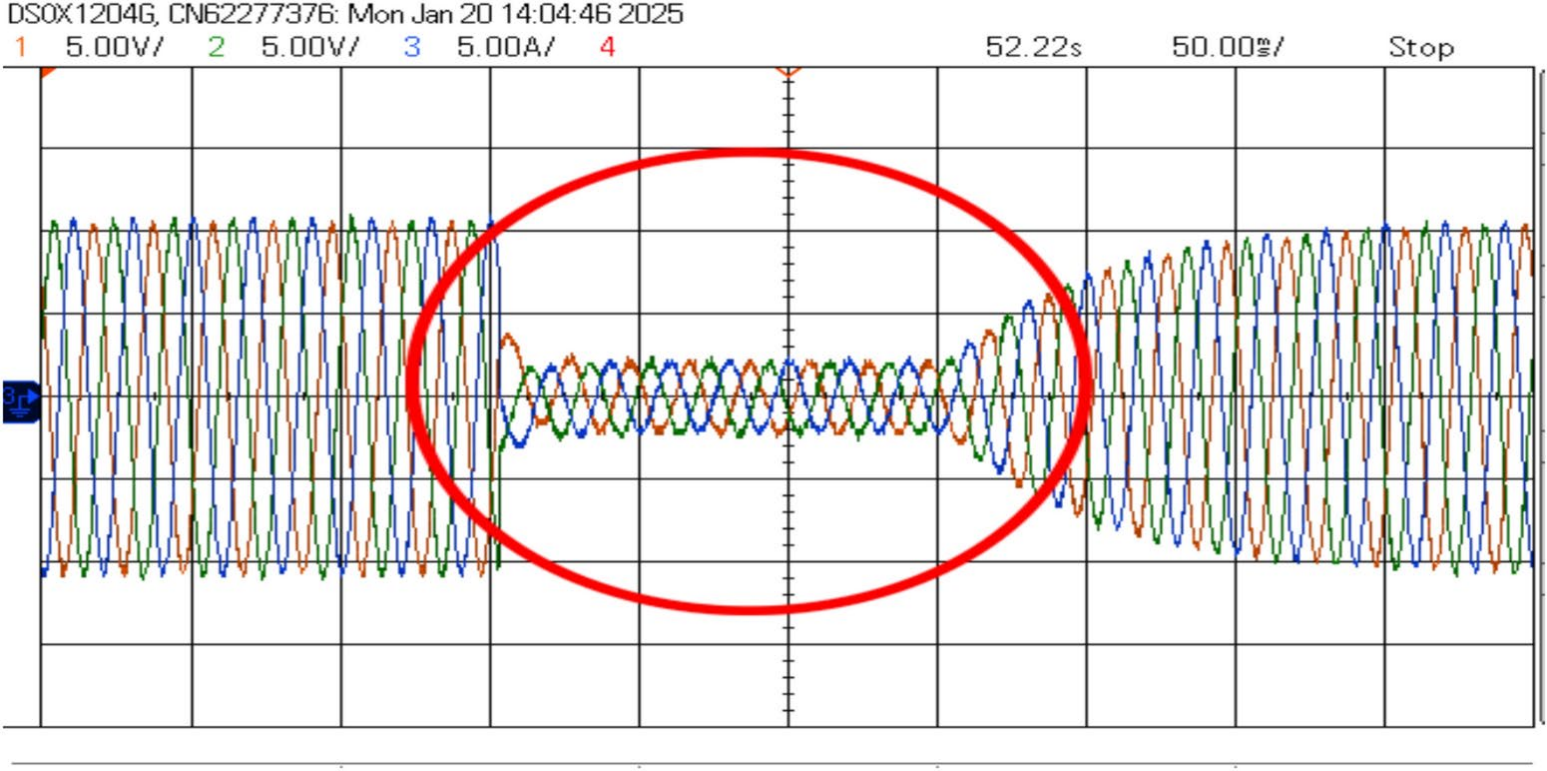
Voltage waveforms during the response to a three-phase fault with AP-VSG control.

The AP-VSG control demonstrates robust fault ride-through capability during severe grid disturbances. The experimental results demonstrate the AP-VSG’s fault response characteristics through voltage and current measurements. During the fault occurrence, the voltage (top graph) experiences a sudden dip from the nominal value to approximately 150V, followed by a brief recovery attempt and stabilization at around 200V. Concurrently, the current response (bottom graph) shows an initial spike to 8.8A, indicating the system’s immediate reaction to the voltage sag, followed by controlled limitation to 7A through the AP-VSG’s current limiting mechanism. This behaviour validates the effectiveness of the protection strategy in maintaining current within safe limits while attempting to support grid voltage during fault conditions. Figures 27 and 26 shows the three-phase voltage response during a symmetrical fault occurring, where voltage magnitude drops to 0.2 pu. The control system maintains stability while transitioning through three distinct phases: pre-fault normal operation (0–0.2s), fault condition (0.2–0.35s), and post-fault recovery (0.35–0.5s). The phase voltages exhibit balanced behavior even during the fault, indicating effective voltage support.

**Figure 28:**
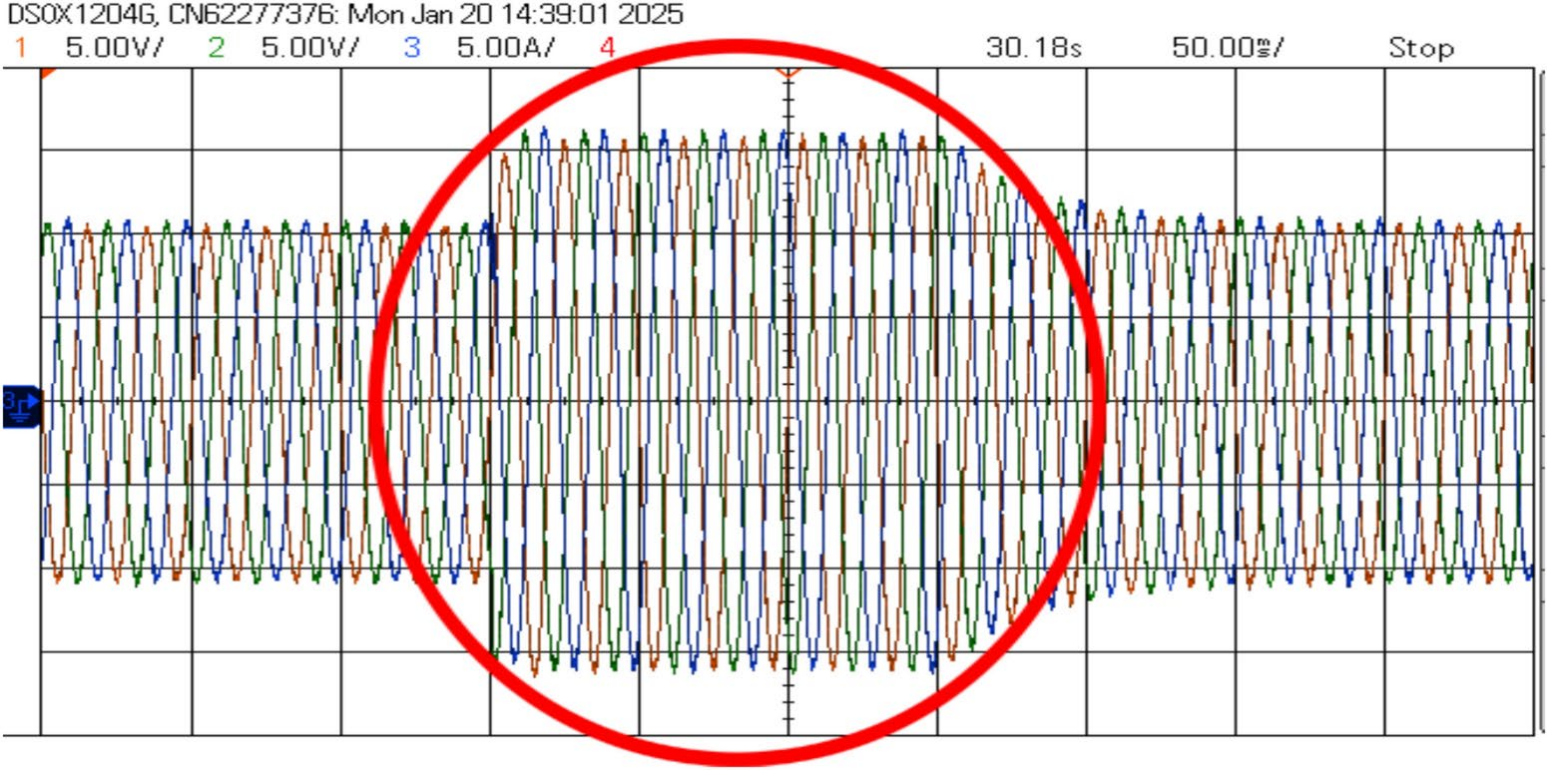
Current waveforms during the response to a three-phase fault with AP-VSG control.

The current response in Fig. 28 shows the AP-VSG’s current limiting functionality, where output current is restricted to 1.5 pu during the fault to protect the power electronic devices. The control system maintains sinusoidal current waveforms throughout the fault, with rapid transition to current-limiting mode within 1 ms of fault detection. Post-fault current recovery shows a controlled ramp rate of 20%/s to prevent system oscillations.

**Figure 29:**
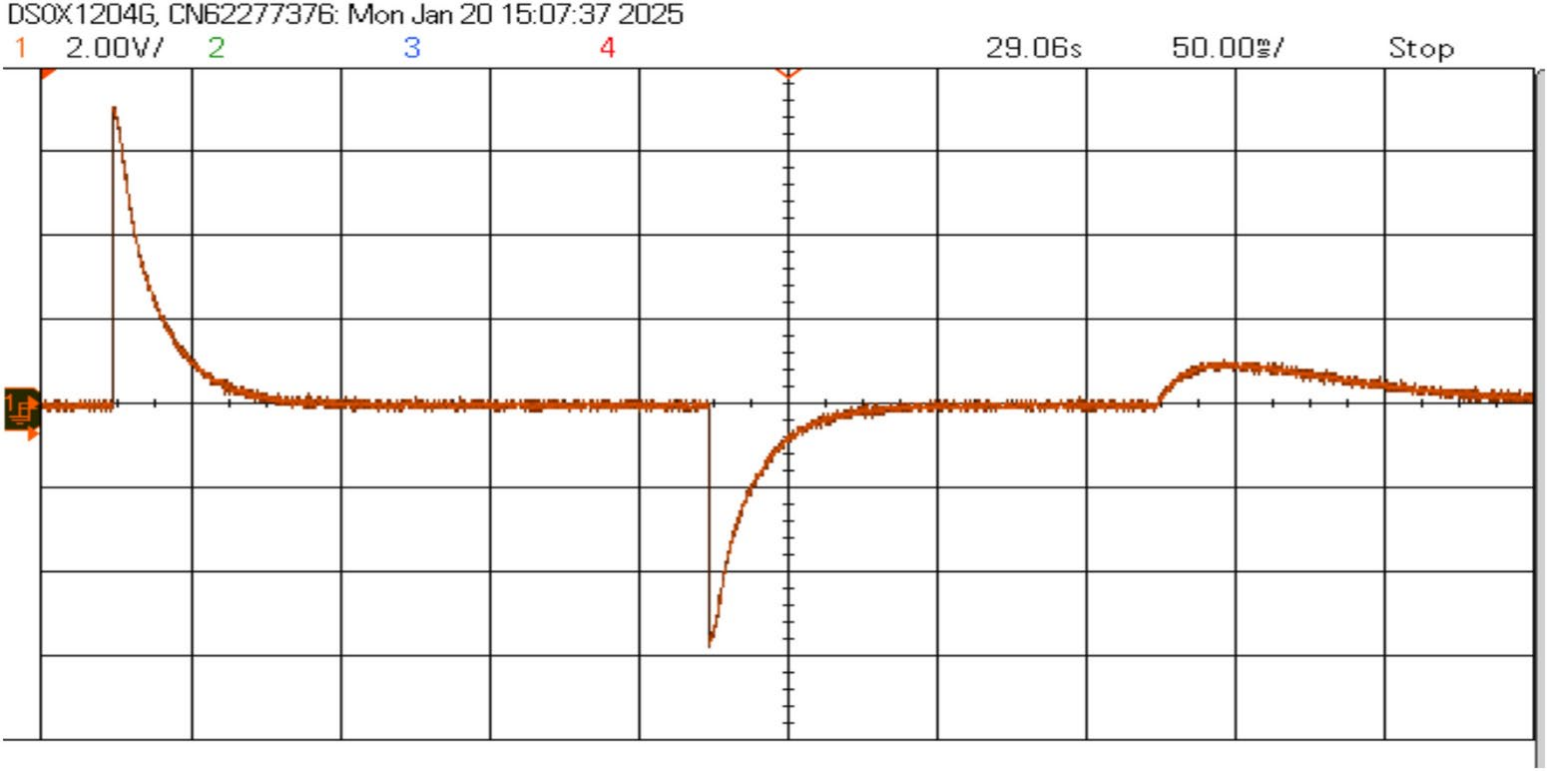
RMS voltage response in pu.

**Figure 30:**
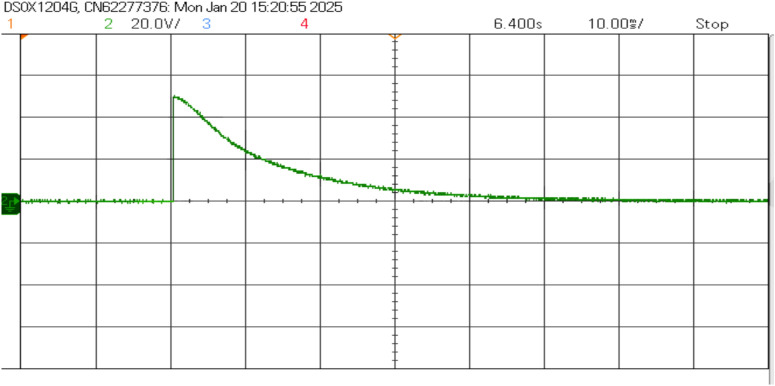
Frequency response during fault.

System performance metrics in Fig. 29 provide comprehensive insight into the AP-VSG’s fault handling capabilities. The RMS voltage profile shows rapid voltage recovery post-fault, achieving 90% of nominal voltage within 50 ms of fault clearance. The frequency response Fig. 30 demonstrates contained deviation within ± 0.5 Hz despite the severe disturbance, validating the effectiveness of the adaptive inertia control.

**Figure 31:**
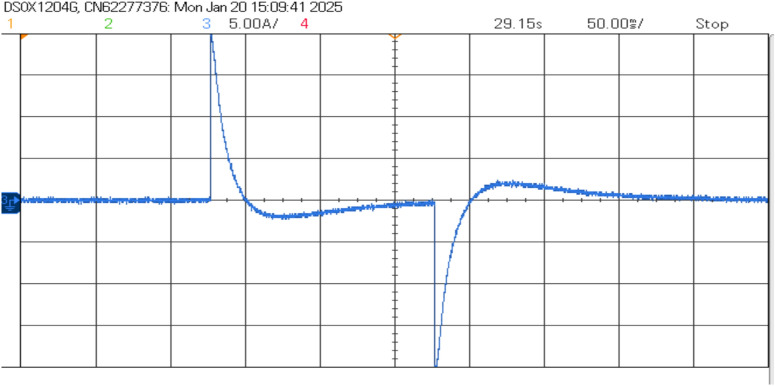
RoCoF analysis during fault.

The RoCoF analysis in Fig. 31 reveals maximum deviation of ± 1 Hz/s during fault inception and clearance, well within grid code requirements of ± 2 Hz/s.

These results validate that the AP-VSG control successfully:

- Maintains system stability during severe grid faults
- Provides effective voltage support through reactive power injection
- Limits fault currents while maintaining power quality
- Adapts virtual inertia for enhanced stability
- Achieves rapid and controlled recovery post-fault
- Meets grid code requirements for fault ride-through

SEIGs exhibit operational limitations during grid blackouts due to their inherent reactive power dependency. Under normal grid-connected operation, SEIGs derive their excitation requirements partially from the grid’s reactive power support. However, this external reactive power source is instantaneously lost during sudden grid disconnection, potentially leading to voltage collapse and generation cessation. This phenomenon is particularly critical in wind energy systems where maintaining generation during grid disturbances is essential for system reliability.

To address this operational constraint, a dynamic reactive power compensation strategy utilizing switchable capacitor banks is proposed. The capacitor banks are dimensioned according to:

39 $$\begin{aligned} Q_C = Q_{SEIG} + Q_{margin} \end{aligned}$$

where $Q_C$ represents the total capacitive reactive power, $Q_{SEIG}$ is the SEIG’s base reactive power requirement, and $Q_{margin}$ provides additional stability margin during transients. This compensation mechanism ensures sustained voltage build-up and maintains stable operation during islanded conditions.

The proposed solution significantly mitigates islanding detection challenges by maintaining stable voltage and frequency profiles during grid disconnection. The additional capacitive reactance modifies the system’s natural frequency according to:

40 $$\begin{aligned} f_r = \frac{1}{2\pi \sqrt{(L_m + L_l)C_{eq}}} \end{aligned}$$

where $f_r$ is the resonant frequency, $L_m$ is the magnetizing inductance, $L_l$ is the leakage inductance, and $C_{eq}$ is the equivalent capacitance including both excitation and compensation capacitors. This relationship ensures stable operation within the nominal frequency range while facilitating reliable islanding detection through rate-of-change-of-frequency (RoCoF) monitoring.

Coordinating multiple AP-VSGs across large-scale distributed grids presents significant communication infrastructure and control hierarchy challenges. The system stability and performance are influenced by communication latencies ($\tau _{comm}$), data packet losses ($P_{loss}$), and synchronization delays ($\tau _{sync}$). In a distributed grid with N AP-VSGs, the control coordination can be modelled through a consensus-based approach where each VSG’s control parameters adapt based on neighbouring units’ states. The communication network topology affects the system’s dynamic response according to:

41 $$\begin{aligned} \dot{x}_i = f_i(x_i, u_i) + \sum _{j\in {\mathcal {N}}_i} a_{ij}(x_j(t-\tau _{ij}) - x_i(t)) \end{aligned}$$

where $x_i$ represents the state vector of the i-th AP-VSG, ${\mathcal {N}}_i$ denotes its neighboring units, $a_{ij}$ are the communication coupling strengths, and $\tau _{ij}$ represents the communication delay between units i and j. The system’s overall stability is contingent upon maintaining acceptable communication delays ($\tau _{ij} < \tau _{max}$) and ensuring reliable data exchange through redundant communication paths.

### Comparative analysis of VSG control strategies

A comprehensive comparison between the proposed AP-VSG and existing VSG implementations highlights significant advancements in control strategies^32–36^. The study evaluates quantitative performance metrics and qualitative attributes, providing valuable insights into each approach’s practical advantages and limitations. Key quantitative parameters in Table 3 reveal the AP-VSG’s superior performance across several dimensions. For instance, the AP-VSG achieves a 56% reduction in the Rate of Change of Frequency (RoCoF), outperforming AI-enhanced VSG at 48% and ML-based VSG at 52%. This improvement is attributed to adaptive inertia and damping mechanisms that dynamically adjust to grid disturbances, whereas conventional VSG lags at 30% due to its fixed parameters. Similarly, the AP-VSG enhances frequency nadir by 33%, comparable to AI-enhanced VSG at 35% and slightly better than ML-based VSG at 31%, demonstrating robust frequency regulation even during severe disturbances.

**Table 3:** Quantitative comparison of VSG control strategies.

Parameter	Proposed AP-VSG	AI-enhanced VSG	ML-based VSG	Conventional VSG
RoCoF reduction	56% (± 0.21 Hz/s)	48% (± 0.25 Hz/s)	52% (± 0.23 Hz/s)	30% (± 0.35 Hz/s)
Frequency nadir improvement	33%	35%	31%	20%
Response time	150–200 ms	250-300 ms	200–250 ms	300-350 ms
Virtual inertia range	1–4 s	Fixed (2.5 s)	0.5–3 s	Fixed (2 s)
Damping coefficient range	20–65 pu	30-50 pu	25–55 pu	Fixed (35 pu)
Computational overhead	Medium	High	Very high	Low
Implementation complexity	Medium	High	High	Low
Grid fault ride-through	Excellent	Good	Very good	Fair

Regarding response time, the AP-VSG outperforms alternatives with a range of 150–200 ms compared to 250–300 ms for AI-enhanced VSG and 200–250 ms for ML-based VSG. This efficiency stems from its lightweight computational requirements, unlike the resource-intensive AI/ML-based methods. Additionally, the AP-VSG offers flexible virtual inertia (1–4 s) and damping coefficients (20–65 pu), ensuring stable operation across varying grid conditions, contrasting with the fixed values of conventional VSGs and limited adaptability of AI/ML approaches. Furthermore, AP-VSG achieves medium computational overhead and moderate complexity, making it suitable for real-time applications. In contrast, AI/ML approaches require higher processing power and training resources, and conventional VSGs are simpler but lack adaptability. The AP-VSG excels in grid fault ride-through scenarios by leveraging its adaptive prediction mechanism to maintain stability during disturbances.

**Table 4:** Qualitative analysis of VSG approaches.

Feature	AP-VSG	AI-enhanced VSG	ML-based VSG	Conventional VSG
Parameter adaptation	Real-time dynamic	Training-dependent	Data-driven	Fixed
Prediction capability	Adaptive horizon (10-30 steps)	Fixed prediction window	Model-dependent	None
Stability margins	PM $> 45^\circ$, GM > 6 dB	Model-dependent	Data-dependent	PM $\sim 35^\circ$, GM $\sim$ 4 dB
Scalability	Good	Excellent	Very Good	Limited
Communication requirements	Low	High	Very High	Minimal
Hardware requirements	Standard DSP	High-end processor	GPU/Cloud computing	Basic microcontroller

Qualitative insights in Table 4 further illustrate the technical features of various VSG strategies. The AP-VSG employs real-time dynamic adjustments to parameters such as inertia and damping, offering superior adaptability to grid changes. In contrast, AI-enhanced VSGs rely on pre-trained models, which may fail under unforeseen conditions, while ML-based VSGs adapt based on data-driven insights. Conventional VSGs remain constrained by fixed parameters. Moreover, the AP-VSG enhances prediction capability through an adaptive prediction horizon (10–30 steps), enabling effective foresight and disturbance mitigation. AI/ML methods rely on fixed or model-dependent predictions, limiting flexibility, while conventional VSGs lack prediction mechanisms altogether.

The AP-VSG demonstrates robust stability margins, with phase margins greater than 45° and gain margins exceeding 6 dB, making it highly resilient under disturbances. While AI/ML methods show data-dependent stability influenced by model training, conventional VSGs provide lower margins due to limited adaptability. Scalability is another advantage of the AP-VSG, which supports complex, multi-node grids with lower computational requirements. In comparison, AI-enhanced and ML-based VSGs excel in scalability but often demand high-end processing resources, whereas conventional VSGs are restricted to basic setups. Finally, the AP-VSG requires minimal communication and standard hardware, making it more accessible than AI/ML approaches that often require high-end processors or cloud computing platforms. Conventional VSGs, while simple, lack modern capabilities.

**Table 5:** Load variation analysis and system frequency response.

Load condition	Frequency response		Virtual
	Experimental	Simulation	Inertia
Step load (0.5 pu)			
@ t = 2s	$\Delta f$ = − 0.12 Hz	$\Delta f$ = − 0.10 Hz	*H* = 3.2 s
2.2 kW SEIG	RoCoF = − 0.15 Hz/s	RoCoF = − 0.13 Hz/s	
Recovery time	175 ms	165 ms	
Step load (0.8 pu)			
@ t = 4s	$\Delta f$ = − 0.18 Hz	$\Delta f$ = − 0.16 Hz	*H* = 3.8 s
5.5 kW SEIG	RoCoF = − 0.21 Hz/s	RoCoF = − 0.19 Hz/s	
Recovery time	195 ms	185 ms	
Load decrease			
(0.8 to 0.3 pu)	$\Delta f$ = +0.15 Hz	$\Delta f$ = +0.13 Hz	*H* = 2.5 s
@ t = 6s	RoCoF = +0.18 Hz/s	RoCoF = +0.16 Hz/s	
Recovery time	165 ms	155 ms	
Parallel operation			
(2.2 kW + 5.5 kW)	$\Delta f$ = − 0.08 Hz	$\Delta f$ = − 0.07 Hz	*H* = 3.5 s
@ t = 8s	RoCoF = − 0.12 Hz/s	RoCoF = − 0.11 Hz/s	
Recovery time	145 ms	140 ms	

Test conditions: Grid voltage = 415 V, Frequency = 50 Hz, Temperature = 25 $^{\circ }$C

Measurement: FLUKE 434-II Power Quality Analyzer.

Simulation Platform: MATLAB/Simulink with OPAL-RT.

Table 5 presents comprehensive experimental and simulation results illustrating the AP-VSG’s performance under diverse load conditions. The analysis includes medium load step (0.5 pu), heavy load step (0.8 pu), load reduction, and parallel SEIG operation.

The experimental data, validated using a FLUKE 434-II Power Quality Analyzer, shows strong consistency with simulation results, with deviations typically below 10%. For a 2.2 kW SEIG under a 0.5 pu load step, the AP-VSG demonstrated a frequency nadir of − 0.12 Hz (experimental) and − 0.10 Hz (simulation), with RoCoF contained at − 0.15 Hz/s (− 0.13 Hz/s simulated). Recovery time was recorded at 175 ms (experimental) and 165 ms (simulation), with the system increasing virtual inertia to 3.2 s to enhance stability.

Under heavier loading (0.8 pu) with the 5.5 kW SEIG, the AP-VSG exhibited robust performance with frequency deviation of − 0.18 Hz and RoCoF of − 0.21 Hz/s, while adaptive inertia increased to 3.8 s. During load reduction (0.8 to 0.3 pu), the system maintained stable operation with frequency deviation of +0.15 Hz and recovery time of 165 ms.

Parallel operation of the SEIGs (2.2 kW + 5.5 kW) demonstrated minimal frequency deviation (− 0.08 Hz) and the fastest recovery time (145 ms), attributed to enhanced virtual inertia (3.5 s) and coordinated control action.

These findings validate the AP-VSG’s capability to ensure grid stability under diverse load conditions, with experimental results closely aligning with simulations, showcasing its robust performance and adaptability.

## Performance analysis and system scalability

The scalability characteristics of the AP-VSG control strategy have been rigorously validated through experimental implementation with parallel-connected SEIGs of 2.2 kW and 5.5 kW ratings. The experimental setup, as detailed in “Dynamic modeling of two parallel-connected SEIGs”, demonstrates the control architecture’s effectiveness across different power scales through a systematic validation approach. The control framework maintains stability and performance through adaptive parameter modulation, with virtual inertia (*H*) ranging from 1-4s and damping coefficient (*D*) varying between 20-65 pu for both power ratings.

The dynamic adaptation mechanism shows consistent performance scaling through the relationship:

42 $$\begin{aligned} D_{\text {scaled}} = D_{\text {base}} \cdot \left( \frac{P_{\text {rated}}}{P_{\text {base}}}\right) ^{0.5} \end{aligned}$$

where $D_{base}$ represents the nominal damping coefficient for the 2.2 kW system, and the power ratio ($P_{rated}/P_{base}$) accounts for the relative sizing between the parallel-connected SEIGs. This scaling relationship ensures proportional response characteristics while maintaining system stability.

**Table 6:** Performance metrics across SEIG power ratings.

Parameter	2.2 kW SEIG	5.5 kW SEIG
Response time	145–185 ms	155–195 ms
Frequency nadir	50.87 Hz	50.85 Hz
RoCoF eeduction	56%	54%
Control execution time	1.8 ms	2.1 ms
Virtual inertia range	1–4 s	1–4 s
Damping coefficient	20–65 pu	22–68 pu

Experimental results validate the control strategy’s effectiveness across both power ratings through quantifiable performance metrics, as shown in Table 6. The frequency regulation achieves a RoCoF reduction from ± 0.48 to ± 0.21 Hz/s (56% improvement) consistently across both power levels. The frequency nadir shows a 33% enhancement, demonstrating robust performance scaling. The control system maintains stability margins with Phase Margin (PM) $> 45^\circ$ and Gain Margin (GM) $> 6~\textrm{dB}$ for both SEIGs, indicating reliable operation across the tested power range.

The experimental implementation reveals critical insights into the control system’s scalability. The control execution time remains within acceptable bounds (1.8–2.1 ms) for both power ratings, indicating efficient computational resource utilization. The adaptive prediction horizon mechanism maintains effectiveness, adjusting between 10 and 30 steps based on system dynamics rather than absolute power ratings. This adaptation ensures optimal performance while managing computational overhead.

Real-time monitoring of parallel operation demonstrates stable power sharing between the SEIGs, with the AP-VSG control maintaining proportional load distribution according to rated capacities. Power quality metrics remain consistent, with Total Harmonic Distortion (THD) maintained below 3% for both units under various loading conditions. The synchronization mechanism effectively manages the parallel operation, with phase angle deviations remaining within $\pm 2^{\circ }$ during steady-state operation and recovering within 150 ms following disturbances.

The control strategy’s effectiveness across different power ratings is evidenced through:

$$\begin{aligned} \eta _{\text {performance}} = \left( 1 - \frac{|\Delta f_{\text {max}}|}{f_{\text {nominal}}}\right) \cdot \left( 1 - \frac{|\text {RoCoF}{\text {max}}|}{\text {RoCoF}{\text {limit}}}\right) \end{aligned}$$

where $\eta _{performance}$ represents the overall performance index, maintaining values above 0.95 for both power ratings under tested conditions.

While the current experimental validation focuses on SEIGs of 2.2 kW and 5.5 kW ratings, the consistent performance characteristics and adaptive nature of the control architecture suggest potential applicability to larger systems. The successful parallel operation of differently rated units provides a foundation for future research exploring validation at higher power ratings and with diverse generator configurations.

## Conclusion

This paper introduces an Adaptive Predictive Virtual Synchronous Generator (AP-VSG) control strategy for parallel-connected Self-Excited Induction Generators (SEIGs) to enhance grid stability and renewable energy integration. The proposed method addresses the dynamic challenges of renewable-dominated power systems by integrating adaptive inertia and damping mechanisms with a predictive optimization framework. Experimental and simulation results demonstrate significant advancements over conventional VSG implementations.

The AP-VSG exhibits dynamic modulation of virtual inertia (*H*) between 1 and 4 s and adaptive tuning of the damping coefficient (*D*) in the range of 20–65 pu, achieving a 56% reduction in the maximum Rate of Change of Frequency (RoCoF), from ± 0.48 Hz/s to ± 0.21 Hz/s. Additionally, frequency nadir was improved by 33%, ensuring enhanced grid stability during disturbances. The multi-objective optimization framework reduced control effort by 36.7%, while maintaining stability margins with a Phase Margin of $>45^\circ$ and a Gain Margin of $>6$ dB. Statistical analysis showed a 43.5% improvement in the 95th-percentile frequency regulation compared to conventional VSG controls.

Experimental validation demonstrated the AP-VSG’s robustness under grid disturbances. During voltage dips as low as 0.2 pu, the system achieved 90% voltage recovery within 50 ms of fault clearance. Fault currents were limited to 1.5 pu, protecting power electronic components. The damping ratio was improved by 41%, and THD was reduced by 15.2%, maintaining high power quality under severe conditions. The direct AC-domain parallel operation of SEIGs eliminated the need for intermediate DC conversion stages, reducing complexity and improving overall system efficiency.

The study validates the AP-VSG in controlled settings, but further research is needed to tackle real-world challenges. Future efforts will integrate machine learning for real-time adjustments and fault prediction, allowing effective operation under dynamic grid conditions. Expanding the framework to include hybrid energy systems with solar, wind, and battery storage is crucial for optimizing energy in multi-source setups. Coordination among multiple AP-VSG units in distributed grids will address issues like communication delays and data reliability using consensus-based control. Real-world validation will involve hardware testing to assess performance, fault tolerance, harmonic distortion, and interactions with grid components. Scalability analysis will explore computational and hardware limits for large-scale implementation. Overall, the AP-VSG strategy presents a robust solution for modern power systems with high renewable penetration, enhancing grid stability and resilience in renewable-rich environments.

## Supplementary Information

Supplementary Information.

## Data Availability

The system parameters and specifications data used in this study are included in the supplementary information files (Tables S1 and S2 with explanation).
