# Supplementary material for: Multiobjective adaptive predictive virtual synchronous generator control strategy for grid stability and renewable integration
Source: Sci Rep. 2025 Mar 18;15:9241. doi: 10.1038/s41598-025-93721-y (PMC11920424; doi:10.1038/s41598-025-93721-y)
Supplement: Supplementary file 1 — Supplementary Information. [file 41598_2025_93721_MOESM1_ESM.pdf]

Supplementary Information:

Advanced Multi-Objective Adaptive-Predictive Virtual Synchronous Generator (AP-VSG) Control Strategy for Enhanced Grid Stability and Renewable Integration

Mrinal Kanti Rajak and Rajen Pudur
Department of Electrical Engineering
National Institute of Technology Arunachal Pradesh, India

1 System Parameters and Performance Specifications

To ensure reproducibility and validate the experimental results, the comprehensive system parameters used in implementing the AP-VSG control strategy for parallel-connected SEIGs are presented below. The parameters are categorized into generator specifications, grid interface requirements, filter design, and control system settings.

Table S1: System Parameters and Specifications

**Parameter**	**Value**	**Unit/Comment**
Generator Specifications		
SEIG-1 Rating	2.2	kW
SEIG-2 Rating	5.5	kW
Excitation Cap-1	12.5	μF
Excitation Cap-2	22.5	μF
Rotor Resistance	2.87, 1.04	Ω
Stator Resistance	3.81, 2.07	Ω
Leakage Reactance	4.45, 5.19	Ω
Magnetizing Reactance	87.010, 45.883	Ω
Base Impedance	86.113, 28.704	–
Grid Parameters		
Base power	5000	VA
Base angular frequency	$2 \cdot \pi \cdot f_b$	ω_b
Grid Voltage	415	V (RMS)
Grid Frequency	50	Hz (f_b)
DC Link Voltage	580	V
Switching Frequency	15000	Hz
Sampling Time	0.002	s
LCL Filter Parameters		
C_f	5	μF
L_g	2.15	mH
L_i	2.45	mH
Control Parameters		
Base Inertia (H_0)	2	s
Max Inertia ($H_{\max}$)	4	s

Parameter	Value	Unit/Comment
Base Damping (D_0)	20	pu
Max Damping ($D_{\max}$)	65	pu
Min Prediction ($N_{\min}$)	10	steps
Max Prediction ($N_{\max}$)	30	steps
k_p	9	PI controller
k_i	1	PI controller

The SEIG parameters were experimentally determined through no-load and blocked rotor tests. The excitation capacitances were selected to ensure rated voltage buildup at nominal speed while maintaining stable operation. The machine parameters differ between the two SEIGs due to their power ratings, reflected in their respective impedance values and magnetizing characteristics.

The grid interface parameters were designed for stable operation at the point of common coupling (PCC). The DC link voltage provides a 40% margin over peak grid voltage for robust control. The LCL filter was designed with a resonant frequency at $0.1f_{sw}$ (1.5 kHz) to attenuate switching harmonics while ensuring stability. The inverter-side inductance is slightly larger than the grid-side inductance to improve current ripple attenuation.

The AP-VSG control parameters were optimized through systematic testing. The virtual inertia range ensures adequate frequency support during disturbances while maintaining stable operation. The damping range provides sufficient oscillation damping without compromising dynamic response. The adaptive prediction horizon adjusts computational load based on grid conditions, with shorter horizons during steady-state and extended prediction during disturbances.

2 Comprehensive System Parameters and Control Specifications

Table S2: System Parameters and Specifications

Parameter	Value	Unit/Comment
**Grid Code Requirements**		
Voltage Tolerance	±10	%
Frequency Tolerance	±0.5	Hz
Maximum THD	<5	%
Power Factor Range	0.95–1.0	–
Voltage Unbalance	<2	%
**LCL Filter Design Parameters**		
Resonant Frequency	1.5	kHz
Damping Resistance	2.5	Ω
Current Ripple Attenuation	40	dB
Filter Cutoff Frequency	750	Hz
Quality Factor	2.5	–
**Advanced Control Parameters**		
RoCoF Limit	±0.5	Hz/s
Frequency Deadband	±0.02	Hz
Power Ramp Rate	20	%/min

Parameter	Value	Unit/Comment
Virtual Impedance (Z_v)	0.1+j0.3	pu
Reactive Power Droop	3–5	%
Phase Margin	45	degree
Gain Margin	6	dB
**Protection Settings**		
Overcurrent Limit	150	%
Under/Over Voltage	0.8/1.2	pu
Under/Over Frequency	47.5/51.5	Hz
Fault Ride Through	150	ms
DC Link Protection	750	V
**VSG Dynamic Parameters**		
Frequency Droop Coefficient	5	%
Voltage Droop Coefficient	3	%
Active Power Control Gain (K_p)	0.5	pu
Reactive Power Control Gain (K_q)	0.1	pu
Virtual Stator Resistance	0.02	pu
Virtual Stator Reactance	0.1	pu
Virtual Field Time Constant	1.5	s
Synchronization Time Constant	0.1	s
**Multi-Objective Control Parameters**		
Frequency Weight (w_f)	0.4	–
Power Weight (w_p)	0.3	–
Voltage Weight (w_v)	0.2	–
Control Effort Weight (w_u)	0.1	–
RoCoF Weight Factor	0.5	–
Stability Weight Factor	0.3	–
**Stability and Protection**		
Phase Margin	45	degree
Gain Margin	6	dB
Maximum RoCoF	±0.5	Hz/s
Frequency Deadband	±0.02	Hz
Voltage Operating Range	0.9–1.1	pu
Frequency Operating Range	47.5–51.5	Hz
Fault Ride Through Duration	150	ms
Maximum Current Limit	1.5	pu
